# A CARD9 deficiency mouse model recapitulates human chronic CNS candidiasis identifying defective monocytic cell responses in immunopathogenesis

**DOI:** 10.1172/jci.insight.176676

**Published:** 2025-05-27

**Authors:** Marija Landekic, Isabelle Angers, Yongbiao Li, Marie-Christine Guiot, Marc-André Déry, Annie Beauchamp, Lucie Roussel, Annie Boisvert, Wen Bo Zhou, Christina Gavino, Julia Luo, Stéphane Bernier, Makayla Kazimerczak-Brunet, Yichun Sun, Brendan Snarr, Michail S. Lionakis, Robert T. Wheeler, Irah L. King, Salman T. Qureshi, Maziar Divangahi, Donald C. Vinh

**Affiliations:** 1 Department of Microbiology & Immunology, McGill University, Montréal, Québec, Canada.; 2 Meakins-Christie Laboratories, Department of Medicine, McGill University Health Centre, Montréal, Québec, Canada.; 3 Infectious Disease and Immunity in Global Health, Research Institute of the McGill University Health Center, Montréal, Québec, Canada.; 4 Department of Pathology, McGill University Health Center, Montréal, Québec, Canada.; 5 Department of Human Genetics, McGill University, Montréal, Québec, Canada.; 6 Fungal Pathogenesis Section, Laboratory of Clinical Immunology & Microbiology, National Institute of Allergy and Infectious Diseases, Bethesda, Maryland, USA.; 7 Department of Molecular & Biomedical Sciences, University of Maine, Orono, Maine, USA.; 8 Department of Medicine, Division of Infectious Diseases, McGill University Health Centre, Montréal, Québec, Canada.

**Keywords:** Genetics, Immunology, Fungal infections, Innate immunity

## Abstract

Human Caspase Recruitment Domain Containing Protein 9 (CARD9) deficiency predisposes to invasive fungal disease, particularly by *Candida* *spp*. CARD9 deficiency causes chronic central nervous system (CNS) candidiasis. Currently, no animal model recapitulates the chronicity of disease, precluding a better understanding of immunopathogenesis. We established a knock-in mouse homozygous for the recurring p.Y91H mutation (Y91H^KI^) and, in parallel to *Card9^-/–^* mice, titrated the intravenous fungal inoculum to the CARD9 genotype to develop a model of chronic invasive candidiasis. Strikingly, CARD9-deficient mice had predominantly CNS involvement, with neurological symptoms appearing late during infection and progressive brain fungal burden in the absence of fulminant sepsis, reflecting the human syndrome. Mononuclear cell aggregation at fungal lesions in the brain correlated with increased MHCII^+^Ly6C^+^ monocyte numbers at day 1 after infection in WT and Y91H^KI^ mice, but not in *Card9^-/–^* mice. At day 4 after infection, neutrophils and additional Ly6C^+^ monocytes were recruited to the CARD9-deficient brain. As in humans, Y91H^KI^ mutant mice demonstrated cerebral multinucleated giant cells and granulomata. Subtle immunologic differences between the hypomorphic (p.Y91H) and null mice were noted, perhaps explaining some of the variability seen in humans. Our work established a disease-recapitulating animal model to specifically decipher chronic CNS candidiasis due to CARD9 deficiency.

## Abstract 2

CARD9 deficiency manifests with candidal brain abscess/meningo-encephalitis. We developed a mouse model recapitulating chronic CNS candidiasis seen in humans and identified defective monocytic-cell responses in immunopathogenesis

## Introduction

Inborn errors of immunity in Caspase Recruitment Domain Containing Protein 9 (CARD9) lead to spontaneous development of invasive fungal disease in humans, particularly of the central nervous system (CNS), with *Candida albicans* (1). CARD9 deficiency is caused by loss-of-function mutations, ranging from complete loss of protein expression to hypomorphic missense mutations, and it is inherited in an autosomal recessive pattern. The recurring c.T271C (p.Y91H) mutation has been reported in 6.7% of patients deficient in CARD9 (2, 3), but how this missense substitution leads to invasive candidiasis is still poorly understood (2, 4).

Commonly used models of candidiasis in mice rely on pharmacologically induced fungal colonization and dissemination, by antibiotic or corticosteroid treatment, use of anticancer agents that suppress the immune system, or a combination of these strategies (5, 6). These preclinical models reflect the human form of iatrogenic invasive candidiasis, providing great insight into acute fungal disease associated with malignancy (7). However, invasive fungal disease in CARD9 deficiency is spontaneous (i.e., in the absence of exogenous immunosuppression) and indolent; thus, the conventionally used experimental approaches do not accurately model this specific human condition. Consequently, these aforementioned mouse models preclude our understanding of CARD9-dependent mechanisms underlying host antifungal defenses, particularly in the CNS. A model of candidiasis that accurately recapitulates human CARD9 deficiency is invaluable to decipher its immunopathogenesis.

CNS involvement has been studied as an extension of multi-organ disease (disseminated candidiasis) in mice with candidemia (8, 9). This latter approach typically uses an inoculum ranging 1 × 10^4^ to 1 × 10^6^ CFU of *C*. *albicans* yeast cells in C57BL/6 mice delivered by i.v. injection. WT mice that succumb to infection at these doses die of progressive sepsis and kidney failure (10, 11); on necropsy, neutrophil-mediated kidney immunopathology is apparent, as is damage to other organs, including the brain (10, 12, 13). These studies have shown that myeloid cells, namely neutrophils, monocytes, and tissue-resident macrophages, are essential for tissue protection, since ablation or inhibition of any of these cells increases mortality in mice (10, 14, 15). Using this model, *Card9^–/–^* mice show that the early neutrophil infiltration into the brain (24 hours postinfection [p.i.]) is essential to control fungal growth, as impaired neutrophil trafficking enhances disease (9). However, since invasive candidiasis associated with human CARD9 deficiency is often chronic in nature, with a median age at onset of 17.5 years (range, 3.5–58 years) (1, 16), and is not typically accompanied by fulminant sepsis, the relevance of these mouse models to understanding the chronic features of the human disease remains unclear. By using WT mice, but infected with a lower fungal inoculum, a recent study has also demonstrated that mononuclear phagocytes, including brain-resident macrophages (microglia) and monocytes, play a key role in limiting tissue fungal burden by forming “fungus-induced glial granuloma” in the absence of neutrophil infiltration (17). Importantly, the immune response of CARD9-deficient mice at lower doses may also diverge from the response reported using the acute sepsis model of candidemia. Therefore, a preclinical system that more closely models the clinical presentation of CARD9 deficiency in humans would provide an experimental platform to better understand the mechanisms by which CARD9 mediates anti-*Candida* immunity, and it could be used for development of therapeutic strategies.

In this study, we used CRISPR/Cas9 technology to generate a knock-in mouse homozygous for the c.T271C (p.Y91H) mutation (Y91H^KI^ mice). By titrating the *C*. *albicans* inoculum to the CARD9 genotype, we established a clinically pertinent CARD9-deficient model of chronic invasive candidiasis that recapitulates distinct clinical and histopathological features of the human CNS disease, including primarily neurological symptoms, occasional skull destruction, and granulomatous-like inflammation during late-stage infection. At a systemic level, the Y91H^KI^ mice phenocopy *Card9^–/–^* mice in terms of survival, dissemination, and tissue fungal burden, confirming that the homozygous p.Y91H mutation is sufficient for fungal disease. At the level of host defense, Y91H^KI^ neutrophil anticandidal functions parallel those of *Card9^–/–^* neutrophils. By tracing the kinetics of both tissue fungal growth and the myeloid response in the brain, we identify the critical role of mononuclear phagocyte responses to fungal infection in the brain. Specifically, our data implicate MHCII^+^Ly6C^+^ monocyte-derived cells (moDC) as early responders to CNS fungal invasion. The development of a CARD9-dependent chronic invasive candidiasis murine model that better emulates human CARD9 deficiency provides a framework to decipher the disease’s immunopathology and potentially direct the development of mechanism-based intervention strategies.

## Results

### Knock-in of the human p.Y91H mutation in mice.

**Figure 1:**
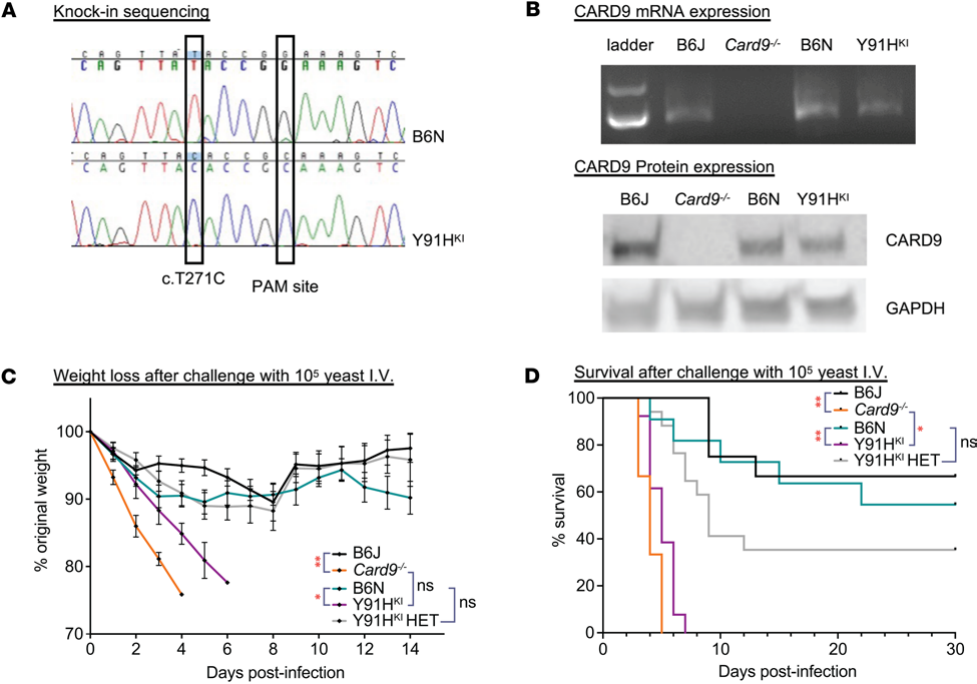
Mice with knock-in of the human p.Y91H mutation phenocopy *Card9^–/–^* mice and are highly susceptible to disseminated candidiasis in vivo. (**A**) Chromatograms of the Sanger sequencing of B6N and Y91H^KI^ mice showing the knock-in and associated silent PAM site mutation. (**B**) RT-PCR showing *Card9* mRNA expression in BMDM and Western blot for CARD9 protein with GAPDH as a loading control. Representative results of 2 experiments shown. (**C**) Weight loss of mice injected via lateral tail vein with 1 × 10^5^ CFU of *C*. *albicans*. Multiple paired 2-tailed *t* tests for significance were performed. *P < 0.05, **P < 0.01 in at least 1 time point. Data are shown as mean ± SEM. (**D**) Kaplan-Meier survival curve of mice injected with 1 × 10^5^ *C*. *albicans* yeast i.v. *P < 0.05; **P < 0.01 by the log-rank test. (**C** and **D**) Three experiments pooled. B6J: C57BL6/J, *n* = 12 mice; *Card9^–/–^*: *n* = 9 mice; B6N: C57BL6/N, *n* = 11 mice; Y91H^KI^: mice homozygous for c.T271C, *n* = 13 mice; Y91H^KI^ HET: mice heterozygous for c.T271C, *n* = 17 mice.

To understand how the p.Y91H mutation in CARD9 predisposes to invasive candidiasis, we used CRISPR/Cas9 to knock in the c.T271C mutation into C57BL/6N (B6N) mice (Supplemental Figure 1A; supplemental material available online with this article; https://doi.org/10.1172/jci.insight.176676DS1). Mice were bred to homozygosity to generate a CARD9^c.T271C/c.T271C^ homozygous knock-in mouse (Y91H^KI^). All genotyping was confirmed by Sanger sequencing (Figure 1A). We initially evaluated *Card9* expression in *Card9^–/–^* (C57BL/6J background; B6J) and Y91H^KI^ BM-derived macrophages (BMDM) by reverse transcription PCR (RT-PCR) (Figure 1B) and Western blot (Figure 1B). B6J and B6N were used as controls for the genetic background of each mutant mouse throughout our investigations. *Card9^–/–^* BMDM show a loss of expression at both the mRNA and protein level. Y91H^KI^ BMDM retained expression of CARD9 at both the transcript and protein levels, consistent with monocytes from patients with CARD9^p.Y91H^ (4, 18). Using flow cytometry, we first performed immunophenotyping of the myeloid compartment of the bone marrow, blood, spleen, brain, and kidney at steady state (Supplemental Figure 1, B–D) and found no differences between the KO, knock-in, or WT mice. These data support the absence of overt myeloid cell deficiencies in patients with CARD9 deficiency (1, 16, 20), indicating CARD9 is redundant for myeloid cell development and homeostasis in mice at steady state (19). Thus, the Y91H^KI^ mouse is a newly developed tool to potentially understand the immunological mechanisms by which CARD9 regulates susceptibility to candidiasis.

### Y91H^KI^ mice phenocopy Card9^–/–^ mice during disseminated candidiasis in vivo.

To begin investigating the in vivo consequences of the p.Y91H mutation, mice were infected i.v. with 1 × 10^5^ yeast cells, a standard model of disseminated candidiasis (9, 11, 13, 14, 21). Mice were then monitored for morbidity (weight loss) and survival (Figure 1, C and D). In this high-dose model, Y91H^KI^ mice succumbed to infection within 7 days. The survival of Y91H^KI^ mice was slightly longer than *Card9^–/–^* mice but did not reach statistical significance. Heterozygous mice displayed an intermediate susceptibility within the 30-day survival experiment. There was no difference in survival between the 2 CARD9 WT mice, showing that any genetic differences between these related strains do not affect survival outcome during infection with high-dose disseminated candidiasis. Thus, Y91H^KI^ mice phenocopied *Card9^–/–^* mice during high-dose infection with respect to acuity, morbidity, and mortality, demonstrating that this protein residue was essential for CARD9 function in mice.

### The p.Y91H mutation results in enhanced fungal growth and susceptibility to disseminated disease.

**Figure 2:**
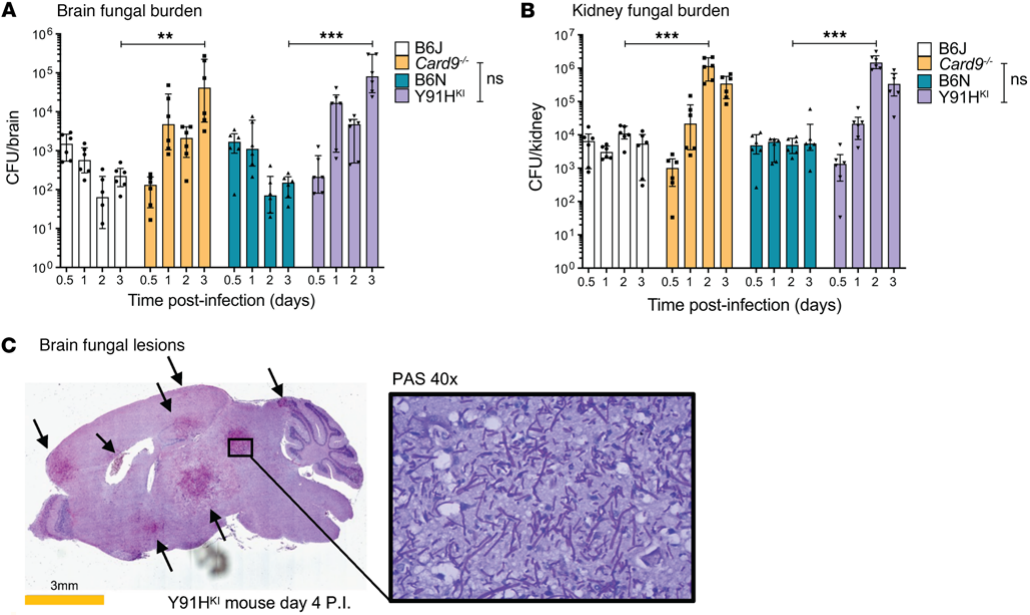
p.Y91H permits enhanced fungal growth in tissue and phenocopies *Card9^–/–^* mice in susceptibility to disseminated disease after high-dose *C*. *albicans* i.v. infection. (**A** and **B**) Dot plots show tissue fungal burden from mice injected via lateral tail vein with 1 × 10^5^ *C*. *albicans* yeast cells. *n* = 6 mice, 2 experiments pooled. Two-way ANOVA with Tukey’s multiple-comparison test. **P < 0.01, ***P < 0.001. Data are shown as mean ± SEM. (**C**) Histopathology showing fungal lesions in the brain of Y91H^KI^ mice after 4 days of infection as for **A**, stained with periodic acid–Schiff. Images were obtained by bright-field microscopy with a 20× objective (whole brain) and 40× objective (zoom) and are representative of 2 experiments. Arrows indicate fungal lesions.

Since lethality during disseminated candidiasis is correlated to kidney fungal burden in WT mice (21), and *Card9^–/–^* mice fail to control fungal growth not only in the kidneys but also in the brain (9, 11), we next compared the kinetics of tissue fungal burden in both kidney and brain (Figure 2). As a control, the fungal burden was also assessed in the spleen, since it is not considered a primary target organ for uncontrolled fungal growth during invasive candidiasis in nonneutropenic hosts (11, 21) (Supplemental Figure 2A). Fungal growth at day 1 p.i. in CARD9-deficient mice is comparable with WT mice, suggesting that initial seeding of *C*. *albicans* from the blood into the tissues was similar (Figure 2, A and B). However, the fungal burden was significantly increased in CARD9-deficient mice in the kidney at day 2 p.i. and in the brain after day 3 p.i. (Figure 2, A and B). Together, these data suggest that the initial hematogenous seeding of *C*. *albicans* into target organs is subsequently restrained in WT mice, whereas Y91H^KI^ and *Card9^–/–^* mice fail to control it, resulting in extensive fungal growth within these tissues.

Histopathology analysis of Y91H^KI^ revealed extensive fungal lesion formation and hyphal filaments (Figure 2C), consistent with published data showing hyphal formation in *Card9^–/–^* mice at the same time point (9). At day 3 p.i., the burden in the spleen of both CARD9-deficient mouse strains significantly increases, and this time point coincides with the onset of mortality (Supplemental Figure 2A). WT mice show no significant increase in spleen burden or mortality within this time frame. Thus, in this high-dose candidiasis model, Y91H^KI^ phenocopy *Card9^–/–^* tissue susceptibility at 3 noncontiguous anatomical locations, suggesting that these mice have comparable defects in antifungal defenses.

### A model of chronic CNS candidiasis caused by CARD9 deficiency.

**Figure 3:**
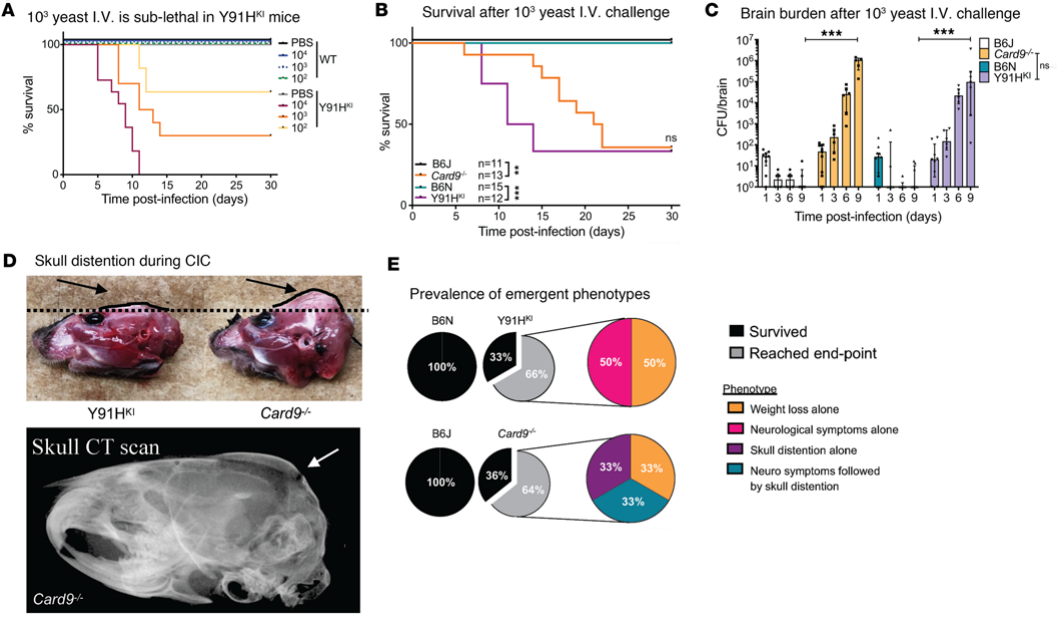
Low-dose i.v. inoculation as a model of chronic CNS candidiasis caused by CARD9 deficiency. (**A**) Kaplan-Meier curve of mice infected with *C*. *albicans*. B6N: *n* = 6–8; Y91H^KI^: *n* = 8–13 mice per group; 2 experiments pooled. (**B**) Kaplan-Meier curve of mice infected with 1 × 10^3^ *C*. *albicans*. *n* = 11–15 per group; 3 experiments pooled. *P < 0.05; **P < 0.01 by the log-rank test. (**C**) Brain burden after 1 × 10^3^ *C*. *albicans* challenge. *n* = 6–11 mice, 3 experiments pooled. Two-way ANOVA with Tukey’s test. ***P < 0.001. Data are shown as mean ± SEM. (**D**) Representative images of skull distention. Gross comparison of the head of a Y91H^KI^ mouse that reached weight loss endpoint and a *Card9^–/–^* mouse that reached endpoint from severe skull distention and morbidity. Arrows and dotted line are visual aids, highlighting the differences in skull morphology. CT scan of a *Card9^–/–^* mouse skull at day 22 p.i. with skull distention is shown. Arrows indicate areas where the skull has lost integrity. (**E**) Prevalence of phenotypes observed in mice from **B**. No symptoms were observed in surviving mice.

Fulminant morbidity and mortality, as well as the disseminated (multiorgan) nature of candidiasis observed in the CARD9-deficient strains using the high-dose approach, poorly models the fungal disease in human CARD9 deficiency, where disease can progress over years with predominant CNS involvement and pauci-involvement of other organs (1, 3, 4). We hypothesized that a fungal dose tailored to the CARD9-deficient state may permit a better understanding of the clinically relevant processes critically regulated by CARD9. Dose response experiments in the Y91H^KI^ mouse revealed an LD_50_ < 1 × 10^3^ yeast cells in a 30-day period, i.e., over 2-log_10_ fold lower than in WT mice (Figure 3A). Unlike the high-dose inoculum (1 × 10^5^ yeast cells), which induced endpoint/terminal disease as early as 3 days p.i., both CARD9-deficient genotypes infected i.v. with the low-dose inoculum (1 × 10^3^ yeast cells) only began reaching endpoints more than 1 week after inoculation (Figure 3B). Weight loss is statistically significant but small in CARD9-deficient mice within the first week of infection (Supplemental Figure 2B); thus, severe morbidity is absent during this time compared with the high-dose model (Figure 1C). These findings indicate that infection with the low-dose *C*. *albicans* inoculum, which results in complete survival of WT mice, causes an indolent form of candidiasis in the CARD9-deficient mice, mimicking the chronicity of fungal disease observed in human CARD9 deficiency.

Dissecting host-pathogen responses in the brain is difficult in the high-dose model of disseminated candidiasis because mice rapidly develop sepsis and die from kidney involvement (Figure 2B) (10–12). We capitalized on our chronic model to investigate these responses. Firstly, we evaluated the kinetics of fungal growth in the brain by CFU assay (Figure 3C). At day 1 p.i., the fungal burden was the same among all groups (Supplemental Figure 2, C and D). WT mice cleared *C*. *albicans* from the brain to the level of detection by day 3 p.i. In contrast, there was an increasing fungal burden in CARD9-deficient mice up to day 9 p.i., when mice began reaching endpoint. *Card9^–/–^* brain burden on day 9 p.i. trends higher than that seen in Y91H^KI^ mice, although the difference was not statistically significant. When comparing tissue fungal burden of Y91H^KI^ mice with *Card9^–/–^* mice in this chronic model (Figure 3C), Y91H^KI^ mice phenocopy *Card9^–/–^* mice in the kinetics and scale of fungal burden in the brain. Overall, these results not only confirm that loss of CARD9 function confers susceptibility to invasive candidiasis but also that it does so by decreasing the threshold of *C*. *albicans* inoculum required to cause disease. Moreover, the comparability in brain fungal burden data between CARD9-deficient mouse genotypes confirms their suitability for the study of chronic invasive CNS candidiasis.

Despite similarities in LD_50_ and tissue involvement with brain proclivity, differences in disease manifestations were observed during the 30-day survival experiment between the Y91H^KI^ and *Card9^–/–^* mice (Figure 3D and Supplemental Videos 1 and 2). Both genotypes displayed overt neurological symptoms, such as altered gait or head tilting late in infection. All mice that displayed symptoms reached the endpoint criteria (Figure 3E). Of the Y91H^KI^ mice that reached endpoint, 50% displayed neurological symptoms. The *Card9^–/–^* mice displayed a more severe phenotype, as 66% of mice that reached the endpoint developed skull deformation (Figure 3, D and E). One-third of the *Card9^–/–^* mice that reached the endpoint displayed neurological symptoms first and then progressed to skull deformation and severe morbidity. Another third had no observed behavioral changes but progressed to skull distention. These necropsy observations were confirmed by CT scan showing that the skull had distended in these mice (Figure 3D). Taken together, these results show that, while both CARD9-deficient mouse strains develop chronic CNS candidiasis with increasing brain fungal burden, *Card9^–/–^* mice develop a more severe phenotype (skull deformity) late in infection.

### CARD9-deficient mice display a progressive inflammatory response in the brain.

**Figure 4:**
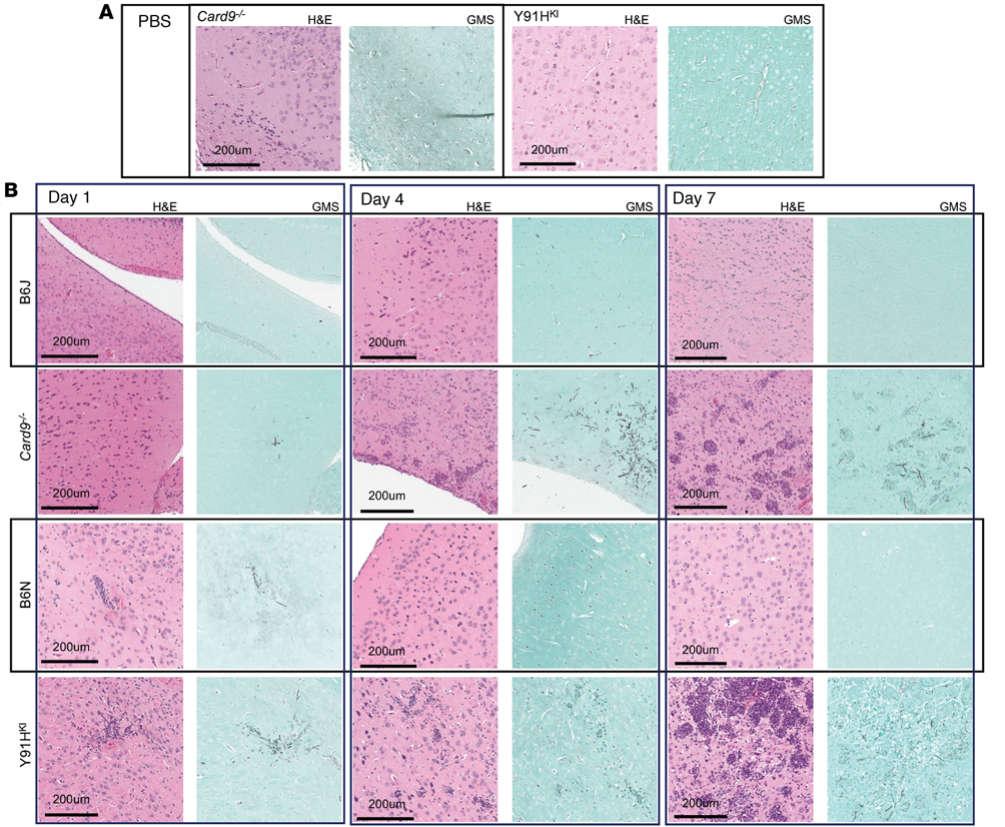
CARD9-deficient mice have increasing inflammatory responses in the brain during chronic invasive candidiasis. Histopathology staining of the brain. H&E (pink) and GMS (green) staining was done on serial sections. Images were obtained by bright-field microscopy with a 20× objective and are representative of 2 experiments. (**A**) PBS-injected controls. (**B**) WT and CARD9-deficient mice at the indicated time points after infection with 1 × 10^3^ *C*. *albicans* yeast cells. Scale bar: 200 μm.

**Figure 5:**
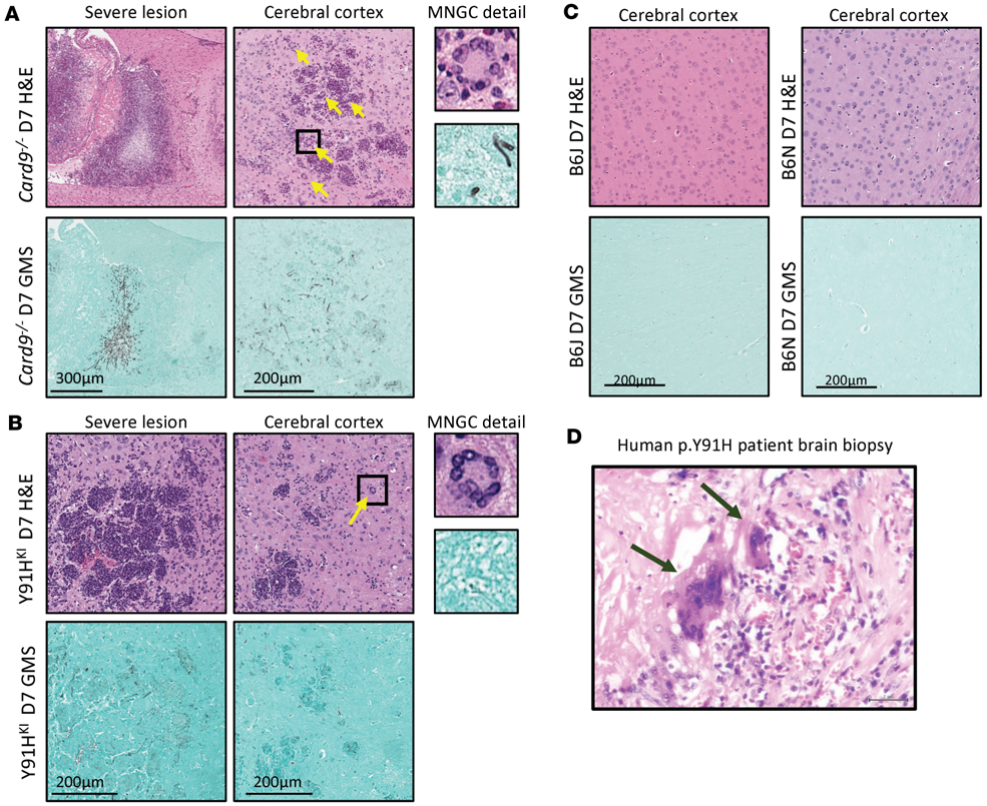
CARD9-deficient mice develop granulomatous inflammation with multinucleated giant cells. (**A**–**C**) Histopathology staining of the brain of CARD9-deficient (**A** and **B**) and WT (**C**) mice at day 7 (D7) p.i. with 1 × 10^3^ *C*. *albicans* yeast cells. GMS and H&E staining were done on serial sections and images shown are matched for anatomical location. Yellow arrows indicate multinucleated giant cells (MNGC). Images were obtained by bright-field microscopy with a 20× objective and are representative of 2 experiments. (**D**) Human p.Y91H patient brain biopsy stained with H&E. Black arrows indicate MNGC. Microphotograph is 400× magnification. Scale bar: 200 μm (**A**–**C**), 2 μm (**D**). “Severe lesion” shows large histopathologic anomalies identified.

To better understand the immunopathogenesis of chronic invasive candidiasis in CARD9-deficient mice, we performed histopathology over the course of disease. Grocott-Gomori’s methenamine silver (GMS) stain for fungus was performed on serial brain sections, along with H&E staining, to correlate areas of fungal lesions with cellular responses (Figure 4, Figure 5, Supplemental Figure 3, and Supplemental Figure 4). No histological differences were observed between noninfected WT and CARD9-deficient mouse brains. One day p.i., mice displayed sparse fungal staining, consistent with the low abundance of CFU detected. Fungal lesions increased in number and size at day 4 and day 7 in CARD9-deficient brain but not in WT mice (Figure 4). As early as day 1 p.i. in Y91H^KI^ mice, inflammatory lesions at sites of fungal staining were observed, becoming more apparent at days 4 and 7 p.i. in both CARD9-deficient strains. From day 4 p.i. onward, as no fungal lesions were detected by GMS in WT samples, images shown were aligned with anatomical locations of fungal lesions from CARD9-deficient samples. By day 7 p.i., robust inflammation and hyphae can be observed in several areas from the ventral striatum to the midbrain and pons.

Detailed analysis of fungal lesions at day 1 p.i. showed yeast and hyphal morphology in both WT and Y91H^KI^ mice, and H&E staining revealed cell aggregates clustering at fungal lesions (Supplemental Figure 3). Fungal lesions detected at day 4 p.i. in CARD9-deficient mice were larger than those observed at day 1 p.i. (Supplemental Figure 4). By day 7 p.i., CARD9-deficient mice showed extensive granulomatous inflammation throughout the brain as well as multinucleated giant cells (Figure 5, A–C). These histological findings were also noted in the brain biopsies of patients deficient in CARD9 bearing p.Y91H (Figure 5D). Interestingly, *Card9^–/–^* mice showed large granuloma formation surrounding a thick fungal mass, as well as multiple smaller granulomata throughout the brain. Granulomata in Y91H^KI^ mice were smaller, focal aggregations of cells distributed throughout the brain.

Collectively, our histopathology data demonstrate hyphal lesions early (day 1 p.i.) in both WT and CARD9-deficient mice; accompanying early inflammatory responses in the WT brain and Y91H^KI^ brain were more apparent. In serial evaluation of WT mouse brain, fungal lesions and perilesional inflammatory foci were no longer seen after day 1 p.i. In contrast, CARD9-deficient mouse brains demonstrated multinucleated giant cells and granulomatous lesions, similar to that seen in humans with CARD9 deficiency. Moreover, the inflammatory lesions appeared more numerous in *Card9^–/–^* mice than Y91H^KI^ mice (Figure 5).

### Inflammatory monocytes accumulate early in the brain.

**Figure 6:**
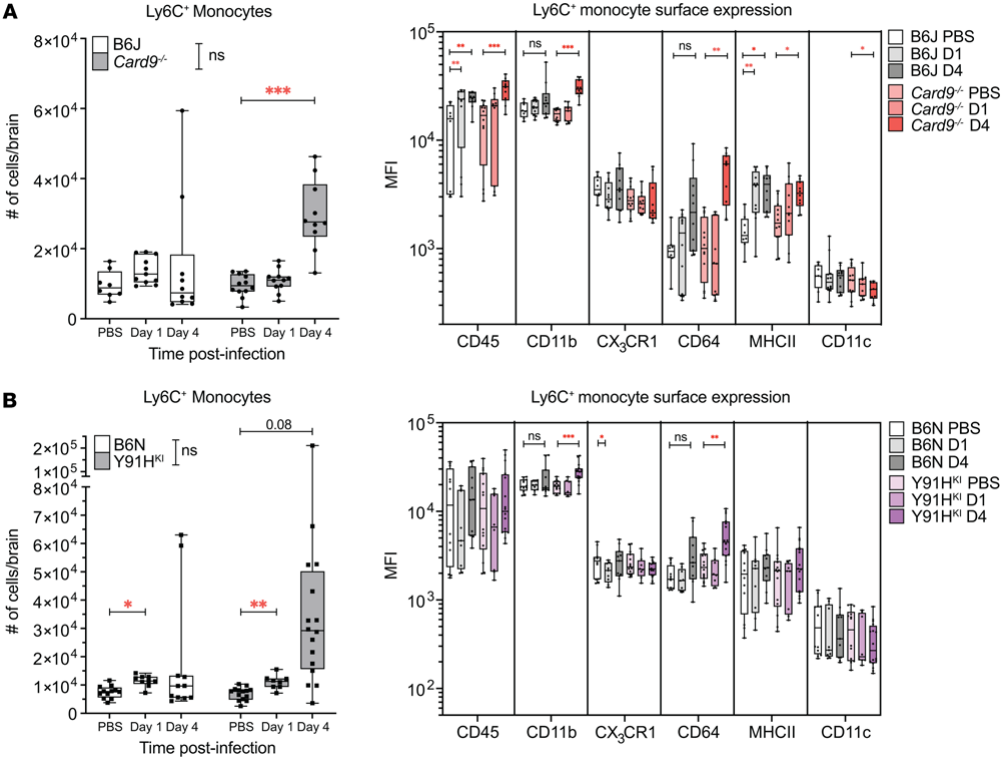
Monocytes and monocyte-derived dendritic cells accumulate in the brain during chronic invasive candidiasis. (**A** and **B**) Box-and-whisker plots showing the number of inflammatory monocytes (CD45^+^CD11b^+^LIN^–^Ly6G^–^Ly6C^+^) and surface marker expression as MFI in the brain of B6J and *Card9^–/–^* mice (**A**) and in B6N and Y91H^KI^ mice (**B**). *n* = 8–16 mice per group, 3 experiments pooled. Median and interquartile range are shown. Two-way ANOVA with Dunnett’s multiple-comparison test done for MFI. *P < 0.05; **P < 0.01; ***P < 0.001.

The identification of multinucleated giant cells and granulomata in this CARD9-deficient chronic invasive candidiasis mouse model, which mirrors what is seen in humans (1, 4, 22) (Figure 5), implicates a functional defect in monocytic responses. Thus, we used this model to define the monocyte responses in CARD9 deficiency. We characterized the early myeloid response in the brain by flow cytometry (Supplemental Figure 5). The number of Ly6C^+^ inflammatory monocytes increased by 35% in WT B6J mice at day 1 p.i. compared with PBS controls, while there was only a 14% increase in *Card9^–/–^* mice; however, neither reached statistical significance (Figure 6A). In contrast, both WT B6N and Y91H^KI^ mice had a significant increase (54% and 38% compared with PBS controls, respectively) in the number of inflammatory monocytes in the brain at day 1 p.i. (Figure 6B), consistent with our histopathology data showing cell aggregations at fungal lesions as early as day 1 on the B6N background (Supplemental Figure 3). In both CARD9-deficient mouse strains, there was an influx of Ly6C^+^ monocytes to the brain by day 4 p.i. that was not observed in WT mice (Figure 6, A and B), coinciding with the divergence of brain fungal burden between CARD9-deficient (increasing) and WT mice (decreasing) (Figure 3C).

We next investigated the expression of surface markers on monocytes in the brain and found a significant increase in markers associated with activation (CD45 and MHCII) at day 1 p.i. on WT B6J cells but not *Card9*^–/–^ monocytes (Figure 6, A and B). *Card9^–/–^* monocytes had increased levels of monocyte activation markers only at day 4 p.i., including CD45, CD11b, CD64, and MHCII. WT B6J monocytes remained activated at day 4 p.i., even as their absolute numbers began to decrease. While WT B6N monocytes only showed a trend toward activation, Y91H^KI^ mice showed a significant increase in CD11b and CD64 expression, suggesting an active monocyte response by day 4 p.i. in this background as well.

**Figure 7:**
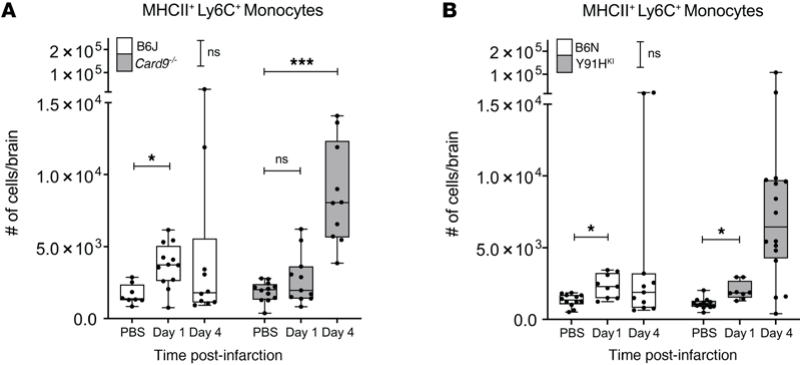
Monocyte-derived MHCII^+^ cells accumulate in the brain during chronic invasive candidiasis. (**A** and **B**) Box-and-whisker plots showing the number of MHCII^+^ inflammatory monocytes (CD45^+^CD11b^+^LIN^–^Ly6G^–^Ly6C^+^MHCII^+^) in the brain of B6J and *Card9^–/–^* mice (**A**) and of B6N and Y91H^KI^ mice are shown (**B**). *n* = 8–16 mice per group, 3 experiments pooled. The median and interquartile range are shown. Two-way ANOVA with Tukey’s multiple-comparison test. *P < 0.05; **P < 0.01; ***P < 0.001.

Since there were significantly higher monocyte numbers on the B6N background at day 1 p.i., but evidence of a higher activation status on monocytes in the WT B6J mice, we next investigated the number of Ly6C^+^MHCII^+^ moDC specifically, as this population is known to be essential for licensing neuroinflammatory responses in other models (23) (Figure 7). Indeed, both WT mouse strains had significantly higher MHCII^+^ moDC in the brain at day 1 p.i. compared with sham-infected control groups (Figure 7). Y91H^KI^ mice also had increased MHCII^+^ moDC at day 1 p.i., while the number in *Card9^-/–^* mice was not significantly increased relative to sham control. These data support a role for activation and differentiation of Ly6C^+^ monocytes during the early responses to *Candida* in the brain and implicate the level of CARD9 functionality (intact in WT, hypomorphic in Y91H^KI^ mice, and absent in the KO) in the regulation of these responses.

Microglia are the most abundant immune cell in the brain and are known to be important in anti-*Candida* resistance during infection in WT mice (11, 17). Thus, we next asked if microglia showed markers of activation, such as CD45, in CARD9-deficient mice. Microglia indeed showed increased CD45 at day 4 p.i. in both CARD9-deficient strains but not WT mice, consistent with their activation during infection at this time point. Additionally, microglia on the WT B6J background showed an increase in cell number on day 1 p.i., which was not observed in the B6N background (Supplemental Figures 6 and 7). Investigation of the activation of other mononuclear phagocytes, including Ly6C^lo^ monocytes, macrophages, and conventional dendritic cells (cDC) revealed signatures of activation by day 4 p.i. but no consistent pattern across the different groups of mice (Supplemental Figures 6–8). There was no increase in eosinophils, NK cells, T cells, or B cells observed in any genotype up to day 4 p.i. (Supplemental Figure 8).

Collectively, these findings demonstrate a localized immune response in the brain of WT mice by day 4 p.i., characterized by the absence of the large influx of peripheral immune cells seen in high-dose models, particularly neutrophils. Moreover, they reveal an early mononuclear phagocyte response in the brain during CNS candidiasis that is dependent on CARD9 signaling. Specifically, the findings demonstrate the early recruitment and engagement of Ly6C^+^ monocytes, a process that is significantly delayed in CARD9-KO mice. Interestingly, despite this initial delay, the monocyte response in CARD9-deficient mice eventually becomes larger in scale compared with that observed in WT mice.

As early monocyte responses in the brain were demonstrated to be important in the high-dose model of candidiasis using *Ccr2^–/–^* mice, which lack circulating monocytes at steady state and show significant delays in monocyte egress from the bone marrow during infection (24), we assessed the importance of our observed monocyte response by evaluating *Ccr2^–/–^* mice infected with low-dose *C*. *albicans*. Indeed, we show that by day 3 p.i., the percentage of *Ccr2^–/–^* mice with detectable fungal burden in the brain begins to diverge from WT controls (Supplemental Figure 9). By day 9 p.i., 71% of infected *Ccr2^–/–^* mice have detectable fungal burden, while only 33% of either WT genotypes do. Additionally, the median fungal burden in *Ccr2^–/–^* mice is 2 log-fold higher than the median of WT mice. These findings suggest that, in the setting of a CARD9-intact background, functional monocytic responses are important in mitigating CNS candidal disease.

**Figure 8:**
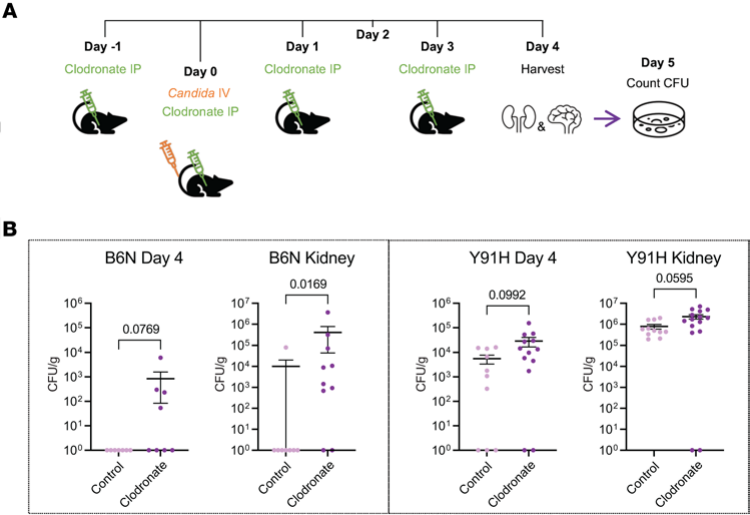
Mononuclear phagocytes are necessary for antifungal immunity in Y91H CARD9 deficiency. (**A**) Schematic of clodronate regime and infection. Mice were treated with control or clodronate as shown and infected with 1 × 10^3^ *C*. *albicans* on day 0. Brain and kidney were harvested 4 days p.i. for enumeration of CFU. (**B**) Brain and kidney fungal burden on day 4 p.i. Data were analyzed using the Mann-Whitney *U* test, and the *P* value is indicated. *n* = 8–15 mice per condition, 3 experiments pooled. Data are shown as mean ± SEM.

To directly assess the role of the monocytic response observed in our chronic infection model, we used liposomal clodronate to deplete mononuclear phagocytes (Figure 8). WT mice of both genotypes were able to clear the fungus from the brain and kidney when treated with liposome vehicle alone, but they had detectable fungal burden in both tissues when treated with clodronate (Figure 8 and Supplemental Figure 9); this finding is consistent with that previously reported using clodronate-based depletion in a high-dose model (14) and confirms our *Ccr2^–/–^* data on the importance of mononuclear phagocytes in this chronic invasive candidiasis model. CARD9-deficient mice did not clear the infection in the liposomal control treatment and showed fungal burden in both the brain and kidney. Y91H^KI^ mice treated with clodronate showed higher candidal burden in both the brain and kidney, confirming that monocytes are necessary for antifungal immunity in the context of CARD9 deficiency in our low-dose model and suggesting that Y91H^KI^ monocytes are at least partially protective, since their depletion leads to higher fungal burden. *Card9^–/–^* mice treated with clodronate developed hypercoagulation resulting in 50% of mice not surviving to day 4 p.i. (Supplemental Figure 9C). Surviving mice showed only a small trend toward higher fungal burden in treated mice verses the liposomal control treatment (Supplemental Figure 9B), likely reflecting the existing delay of monocyte recruitment observed by flow cytometry.

Collectively, these findings demonstrate that monocytic responses are protective, as disruption of mononuclear phagocyte responses exacerbates disease outcomes in CARD9-deficient mice.

### Impaired cellular responses of CARD9-deficient BMDMs.

**Figure 9:**
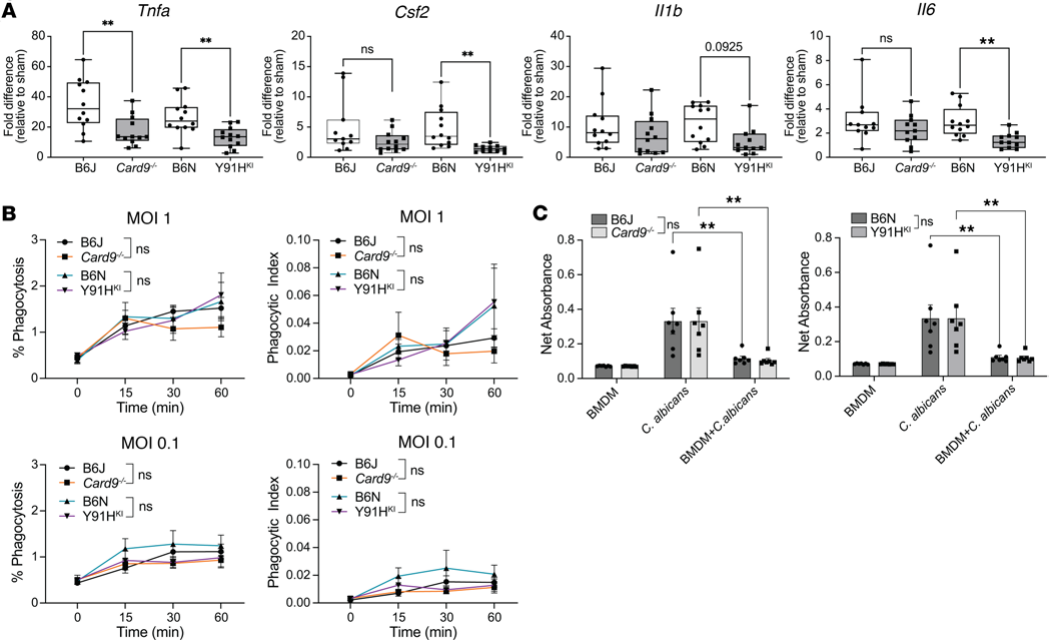
Impaired BMDM cytokine responses to live *C*. *albicans* in CARD9 deficiency. (**A**) BMDM of each genotype were infected in vitro with live *C*. *albicans* yeast at an MOI of 1 for 2 hours. Cytokine expression by qPCR shown. *n* = 12 mice per genotype, 7 experiments pooled. Median and interquartile range are shown. One-way ANOVA test for significance was used. (**B**) BMDM were infected with GFP-tagged yeast cells at indicated MOI. Intracellular yeast enumerated at the times indicated by confocal microscopy. % phagocytosis = (no. of BMDM with internalized yeast/total BMDM counted) × 100. Phagocytic Index = (total no. of engulfed yeast/total no. of counted BMDM) × (no. of BMDM containing engulfed yeast/total no. of counted BMDM) × 100. *n* = 9 mice per genotype, 3 experiments pooled. Data are shown as mean ± SEM. (**C**) BMDM alone, *C*. *albicans* alone, or BMDM infected with an MOI of 0.1 for 6 hours, and an XTT assay was performed. Net absorbance; *n* = 6–7 mice; 3 experiments pooled. Data are shown as mean ± SEM. Two-way ANOVA with Tukey’s multiple-comparison test for significance was used. **P < 0.01.

BMDMs from *Card9^–/–^* mice have been used as a cellular model of CARD9 deficiency to show that CARD9 mediates specific antifungal effector functions, particularly the production of proinflammatory cytokines TNF-α, IL-6, and IL-1β (4, 9, 25). Monocytes derived from p.Y91H humans have also demonstrated the importance of *Csf2* granulocyte-macrophage–CSF (GM-CSF) production by these cells (3, 4). As our data suggest that the early responses of moDC in CARD9 deficiency are important in the context of CNS candidiasis, we infected BMDM with live *C*. *albicans* for 2 hours in vitro and investigated the proinflammatory cytokine response using quantitative PCR (qPCR) (Figure 9A). Both CARD9-deficient genotypes showed impaired *Tnfa* production compared with WT controls. *Card9^–/–^* BMDM produced *Csf2*, *Il1b*, and *Il6* at comparable levels with WT BMDM; however, Y91H^KI^ BMDM showed significantly less production of these important inflammatory mediators.

Because CARD9 functions downstream of pattern recognition receptors (e.g., DECTIN-1) that have been shown to participate in phagocytosis of *C*. *albicans*, we next investigated whether the p.Y91H mutation affected internalization of yeast cells. Using live GFP-tagged *C*. *albicans* and the cell-impermeable dye Calcofluor White, internalized yeast were enumerated over the first hour of infection by confocal microscopy (Figure 9B). We found no difference in percent phagocytosis or phagocytic index (PI) between WT BMDM and both CARD9-deficient strains. This finding confirms previous studies showing that CARD9 is dispensable for phagocytosis of *C*. *albicans* in macrophages and demonstrates that p.Y91 is also not essential for internalization of yeast cells (9, 26).

As BMDM can directly kill internalized yeast cells, we tested the fungicidal activity of Y91H^KI^ BMDM to *C*. *albicans* in vitro using the 2,3-Bis-(2-Methoxy-4-Nitro-Sulfophenyl)-2 (XTT) assay, a measure of cellular metabolism that reflects fungal viability (Figure 9C). CARD9-deficient BMDM, both *Card9^–/–^* and Y91H^KI^, showed no defects in fungal killing when compared with WT controls. These data suggest that the intrinsic capacity of these macrophages to kill phagocytosed yeast cells in vitro is not impaired by CARD9 deficiency.

### Neutrophils respond to C. albicans in the brain in CARD9 deficiency.

**Figure 10:**
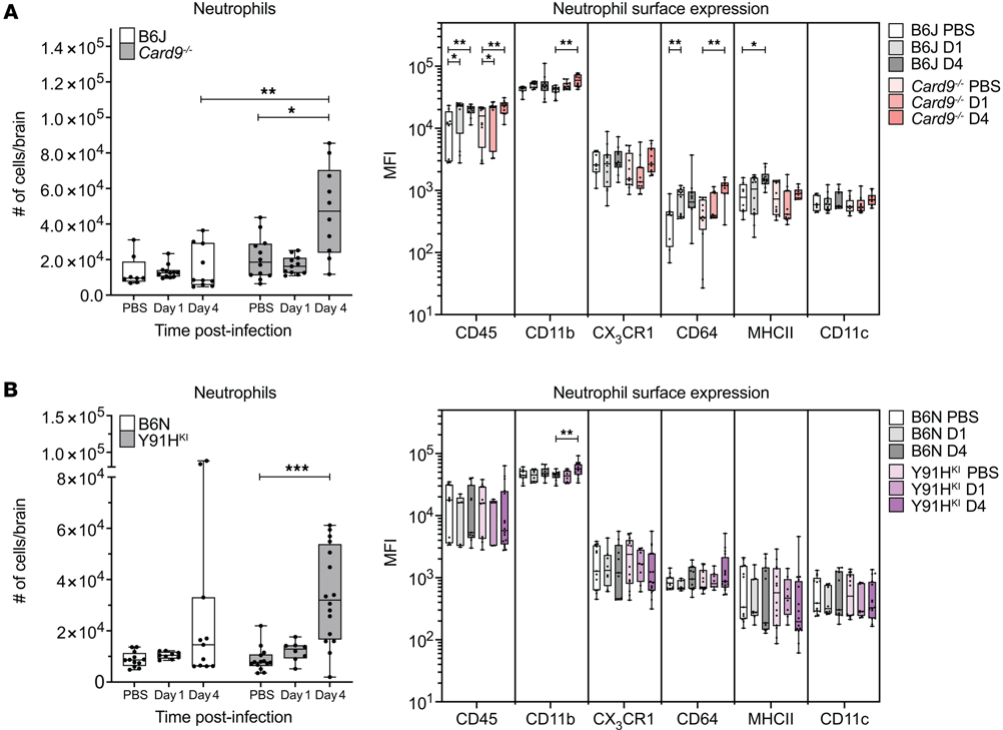
CARD9-deficient mice mount a neutrophil response in the brain during chronic invasive candidiasis. (**A** and **B**) Box-and-whisker plots showing the number of neutrophils (CD45^+^CD11b^+^LIN^–^Ly6G^+^) and surface marker expression as MFI in the brain of B6J and *Card9^–/–^* mice (**A**) and in B6N and Y91H^KI^ mice (**B**). *n* = 8–16 mice per group, 3 experiments pooled. Box-and-whisker plots show the median and interquartile range. Two-way ANOVA with Dunnett’s multiple-comparison test was used for MFI. *P < 0.05; **P < 0.01; ***P < 0.001.

High-dose models of disseminated candidiasis in *Card9^–/–^* mice suggest that impaired neutrophil recruitment to the brain underlies disease (8, 9). Thus, we next investigated the neutrophil response in the brain during the early stages of chronic invasive candidiasis in our low-dose, CARD9-deficient mouse models (Figure 10). WT and CARD9-deficient mice do not recruit neutrophils at day 1 p.i., when we observed MHCII^+^ inflammatory monocytes increase in the brain, but CARD9-deficient mice have significantly higher neutrophil numbers at day 4 p.i. CARD9-deficient neutrophils consistently have higher CD11b expression than at day 1 p.i., suggesting they may be at a higher activation state (Figure 10, A and B). WT mice do not show a significant increase in neutrophil cell numbers and do not have elevated expression of activation markers on neutrophils that are consistent between the WT strains.

Given the presence of neutrophils by day 4 p.i. in our CARD9-deficient mice, we next investigated neutrophil phagocytosis and neutrophil-mediated killing of *C*. *albicans*. Consistent with previous work evaluating CARD9-deficient human and mouse neutrophils (9, 27), ex vivo *Card9^–/–^* neutrophils showed no defect in phagocytosis or killing of serum-opsonized yeast cells but did show impaired killing of nonopsonized yeast (Supplemental Figure 10). Neutrophils from the p.Y91H mouse phenocopied the *Card9^–/–^* neutrophils.

Our current mouse model revealed a neutrophil response to *C*. *albicans* in the brain following infection in CARD9-deficient mice. This finding stands in contrast to observations by others, who reported a limited or minimal neutrophil response (9). By IHC on a brain biopsy of a patient with the p.Y91H mutation, we confirm the modest and scattered presence of neutrophils in situ (Supplemental Figure 11). This suggests that our findings in mice do not inherently conflict with the human data and do not undermine the validity of this disease-recapitulating chronic candidiasis mouse model.

In sum, these data demonstrate that there is some recruitment of neutrophils to the brain during the early stages of CNS candidiasis in the CARD9-deficient mouse, neutrophils are demonstrable in brain biopsy (reflecting later stages) of humans with CARD9 deficiency due to p.Y91H, and *Card9^–/–^* and Y91H^KI^ neutrophils have comparable anticandidal functions.

## Discussion

Human CARD9 deficiency predisposes to invasive fungal infections in the absence of systemic disease, antibiotic or corticosteroid use, cancer, or trauma. The invasive candidiasis of CARD9 deficiency is most commonly caused by *C*. *albicans*, chronic in nature, and late in disease onset and has an unexplained predilection for the CNS. Understanding the immunologic basis by which loss of CARD9 function leads to such a unique syndrome has been hampered by the lack of experimental models that recapitulate these key features.

Here, we first generated mice homozygous for the recurring p.Y91H human mutation. Using a standard high-dose model of candidemia, the Y91H^KI^ mouse phenocopies the *Card9^–/–^* mouse with respect to acuity, morbidity, mortality, and tissue fungal burden, relative to WT mice. Interestingly, *Card9^–/–^* mice die slightly earlier than Y91H^KI^ mice, which may be related to the hypomorphic nature of the latter. Altogether, these findings confirm the utility of the Y91H^KI^ mouse to better understand the antifungal role of CARD9. However, the fulminant and disseminated nature of fungal disease with this high-dose strategy does not accurately recapitulate human CARD9 deficiency. By titrating the candidal dose to the CARD9 genotype, we established a model in which mutant mice demonstrate chronicity of infection, overt neurological symptoms with CNS predilection, and histopathological multinucleated giant cells/granulomata surrounding fungal lesions; these features were elicited at an LD_50_ dose that was at least 2 log_10_–fold lower than the standard, high-dose method. At this lower dose, WT mice completely resolved this infection, displaying no morbidity or mortality. In fact, in our approach, skull destruction is uniquely seen in *Card9^–/–^* mice, consistent with the original report on CARD9 deficiency (28), but is not observed in the Y91H^KI^ mouse in the time frame of these experiments, also mirroring what is observed clinically (3, 4). These aggregated features, which are more aligned with the distinctive clinical features of the human syndrome and are not observed in the high-dose strategy, supports the relevance of this mouse model to define the fungal immunopathophysiology of CARD9 deficiency. It also provides a framework for future antifungal studies of other CARD9 mutations.

We then used this CARD9-mutant, chronic invasive candidiasis model to begin to decipher the immunopathology of CNS candidiasis. Because of previous work demonstrating impaired monocyte and neutrophil responses in humans (3, 4, 9, 27), and our findings of granulomata in CARD9-deficient mice and humans, we focused on early myeloid responses. Our results demonstrate CARD9-KO mice have a delay in the accumulation of activated Ly6C^+^MHCII^+^ monocytes in their brain early in infection, but both CARD9-deficient mice have an abnormal accumulation of these cells by day 4 p.i., corresponding to an increased brain fungal burden histologically, preceding their typical clinical endpoint starting at approximately day 9. Ly6C^+^ monocytes in the brain that upregulate MHCII can license neuroinflammation in models of sterile inflammatory disorders, such as EAE, a mouse model of multiple sclerosis (23). The increase in Ly6C^+^ MHCII^+^ moDC in the brain of infected WT mice is observed at day 1 p.i., implicating this cell type among the earliest responses to candidal brain invasion. These data suggest that CARD9 may be essential for the recruitment or functional differentiation of Ly6C^+^ monocytes in the infected brain, a response that preceded neutrophil influx as fungal burden began to increase in CARD9-deficient mice. Our results are consistent with the observations of Wu et al. showing WT mice that developed transient cerebritis after *C*. *albicans* challenge relied on microglia, in the presence of monocytes but not neutrophils, to clear the fungus (17). We have therefore identified an early, asymptomatic window of time in the pathogenesis of CNS candidiasis associated with a CARD9-dependent activation and influx of Ly6C^+^ monocytes.

The abnormal recruitment of monocytes observed, along with histopathological evidence of multinucleated giant cells/granulomata, may reflect a mechanism for fungal control; on the other hand, they may also be deleterious and cause immunopathology. To distinguish between these 2 possibilities, we showed, through the use of a *Ccr2^–/–^* mouse (which lack circulating Ly6C^+^ monocytes but are CARD9-intact; ref. 24), that these mice could not clear the fungus from their brains over the course of infection, supporting the premise that influx of Ly6C^+^ monocytes are protective for CNS candidiasis. We complemented this evaluation with clodronate-based monocyte depletion experiments in our CARD9-mutant mice, demonstrating that their loss of this response enhanced fungal growth. Collectively, these findings support an important role for mononuclear phagocytes in mitigating CNS candidiasis and show that the abnormal monocytic response observed in the CARD9-deficient mice are at least partially functional and protective. That these monocytic responses are ultimately unable to control candidal growth may underlie the chronic nature of their CNS infection.

While Y91H^KI^ mice can recruit monocytes in the brain at the same level as WT mice, and while our clodronate data confirm their protective nature, our in vitro data using BMDM as a model of terminally differentiated monocytes support the premise that some functions of specialized monocytic cells remain functionally impaired. Our BMDM-mediated phagocytosis experiments revealed no differences between genotypes. Likewise, assessment of BMDM-mediated killing of candidal yeast cells exposed no differences. Because impaired proinflammatory cytokine responses, including IL-1β, IL-6, TNF-α, and GM-CSF, to *C*. *albicans* and fungal agonists is a consistent cellular phenotype of CARD9 loss-of-function in human monocytes (4, 9, 27), we evaluated these in our murine BMDM. Both CARD9-deficient genotypes showed impaired *Tnfa* responses, but only Y91H^KI^ BMDM also showed impairment in *Il1b*, *Il6*, and *Csf2* production. The stochastic distribution of infected foci in the brain precluded assessing these functions in situ, so it remains unclear whether these impaired responses occur in vivo within monocytic, macrophagic, and/or microglial cells. As TNF-α is important for granuloma integrity (17, 29, 30), the impaired TNF-α responses shared by CARD9-mutant BMDM may account for the development of multinucleated giant cells/granuloma-like lesions that may not be able to ultimately contain *C*. *albicans*. Clearly, a broader array of cytokine responses, including spatial assessment and additional CNS cells, is needed to better understand this pathology. Nonetheless, we demonstrate for the first time to our knowledge that, when challenged with a given *C*. *albicans* strain, CARD9-deficient mice of different genotypes (null versus hypomorphic p.Y91H) not only have differences in severity of manifestations at the organismal level but also that the immune response and cellular impairments of each may be subtly distinct. Whether there are differences in immunological response to *C*. *albicans* between distinct CARD9 hypomorphic mutations (for example, based on protein location) is speculative, but it may help clarify variations in clinical trajectory among patients who are CARD9 deficient. Moreover, functional differences between CARD9 genotypes, alone or perhaps in conjunction with functional variants in other genes, may account for the enigmatic susceptibility to the other but seemingly ubiquitous fungi seen in various patients deficient in CARD9.

Interestingly, our mouse model shows that the Y91H^KI^ BMDM have impaired *Csf2* (GM-CSF) responses compared with WT counterparts, which was not seen in *Card9^–/–^* mice. It remains uncertain whether this finding indicates that different CARD9 mutations have distinct effects on GM-CSF responses, perhaps accounting for the observed divergent clinical responses to adjunct GM-CSF therapy between patients with *C*. *albicans* CNS infection (3, 4, 9). Alternatively, other factors may be at play. For example, in the 2 patients we reported where it was beneficial, GM-CSF was given after surgical resection (which debulks the fungal burden), whereas in the patient where it did not provide benefit, no surgical resection was performed (3, 4, 31). Thus, it may be that debulking of brain lesions is necessary for GM-CSF to exert a beneficial effect (e.g., by enabling the more feasible neutralization of any newly developing infectious focus). Alternatively, the differential responses to GM-CSF may suggest that the beneficial effect of GM-CSF therapy is exerted outside of the brain. Indeed, with the establishment of this pathogenetically relevant model, we can now specifically address this question, as well as interrogate the effect of different candidal species, different routes of infection, and responses to different fungal genera altogether.

Another enigmatic feature of CNS candidiasis of CARD9 deficiency is the aberrant neutrophil response. Drummond et al. have shown that neutrophil numbers are unexpectedly low, but definitely not absent, in human CARD9 deficiency; that the neutrophil recruitment response in the mouse brain is proportional to the fungal burden in WT mice but insufficient in CARD9-deficient mice when both genotypes have high brain burden; and that *Card9^–/–^* neutrophils have impaired *Candida* killing against the unopsonized yeast form of the fungus (9). The influx of neutrophils in our CARD9-mutant mice at day 4 p.i. prompted us to reevaluate patient samples, where neutrophils were also seen (although they were not the predominant component of the inflammatory response). The discrepancy in the extent of neutrophil response between the CARD9-deficient mice and in patients could be attributed to differences in the timing of sampling, as onset of infection is precisely known in mice, allowing for early sampling, while patient samples are by definition collected later, during symptomatic stages of the disease. The neutrophils from *Card9^–/–^* or Y91H^KI^ had similar functional capacity — namely, intact phagocytosis of both opsonized and unopsonized yeast cells, intact killing of opsonized yeast, and impaired killing of unopsonized yeast, similar to what has been previously shown (9, 27). We could detect no differences in the patterns of neutrophil recruitment or dysfunction between the KO and the Y91H mutant, suggesting that neutrophil dysfunction is unlikely to explain differences in disease severity seen between the 2 CARD9 mutants. Furthermore, eosinophilic infiltration of CNS tissue can be seen in some patients with CARD9 deficiency, as it can in other patients who do not have CARD9 deficiency but who have chronic fungal infections, and indeed 3 of the Y91H^KI^ mice analyzed for flow cytometry showed eosinophil recruitment to the brain by day 4 p.i. (Supplemental Figure 8). Sampling in our model may have been too early in disease to fully appreciate eosinophilic responses during chronic fungal infection. Overall, these findings support a general concept that loss of CARD9 function alters neutrophil recruitment into the CNS space following *C*. *albicans* infection. Further investigation into the kinetics of the neutrophil response in this chronic candidiasis mouse model will help determine how closely it mirrors the human condition.

In summary, by tailoring the fungal challenge to the susceptibility gene, we have established a mouse model of CARD9 deficiency that more accurately recapitulates the human inborn error of immunity at the clinical and histopathological levels, enabling investigations into the immunopathogenesis underlying chronic CNS candidiasis. Our model identifies that loss of CARD9 function associates with impaired tissue control of low fungal burden and cerebral multinucleated giant cells/granulomata, coupled with abnormal influx of Ly6C^+^ moDC and defective BMDM responses, ultimately contributing to indolent infection with late disease. With this approach, we have additionally shown that p.Y91H is indeed detrimental but results in subtle immunologic differences compared with its null counterpart, raising the possibility that different mutations affect CARD9’s loss of function in distinct and consequential ways. More broadly, our experimentally established framework provides a necessary tool to not only pinpoint how loss of CARD9 function mediates fungal disease, but also for the development of mechanism-targeting therapeutic interventions.

## Methods

### Sex as a biological variable.

Experimental findings were confirmed in both sexes. Sex matching within each experiment decreased total statistical variation, and no significant differences between male and female mice were detected for any of the parameters measured. For human samples, sex was not analyzed as a biological variable in this study. Patients were evaluated based on clinical referral patterns, and the potential relevance of sex differences in CARD9-deficient candidiasis remains unknown.

### Mice.

B6J, *Card9^–/–^*, B6N, and Y91H^KI^ mice were age and sex matched within each experiment. Results from male mice aged 7–12 weeks are shown for fungal burden experiments. All other experiments show female mice aged 7–12 weeks unless otherwise specified. B6J, *Card9^–/–^*, and *Ccr2^–/–^* mice were purchased from The Jackson Laboratory. B6N were purchased from Envigo. Y91H^KI^ mice were generated by CRISPR/Cas9 at McGill Integrated Core for Animal Modeling (MICAM) by microinjecting a gRNA (5′ to 3′ direction) (TCTACTACCCTCAGTTATAC) with a single-stranded oligodeoxynucleotide (ssODN) (CAGCGGACAGGCCACAAGGGCTACGTGGCTTTCCTCGAGAGCCTGGAGCTCTACTACCCTCAGTTACACCGCAAAGTCACTGGCAAGGAGCCAGCACGCGTCTTCTCCATGATCATTGgtgagaggcacgggt), which were both purchased from IDT. The gRNA was complexed with the protein Cas9 and microinjected into the pronucleus of the B6N embryo with the ssODN. These embryos were then transferred into pseudo-pregnant CD-1 females to generate potential F0 mice. After pups were born, ear biopsies were taken to screen for the p.Y91H mutation and a founder male was identified and named Card9^em2Sq^. The male founder was outcrossed to WT B6N females, and heterozygous Card9^em2Sq^ F1 mice were subsequently intercrossed to generate homozygous Card9^em2Sq^ mice (Y91H^KI^). All mice were backcrossed to their respective WT strains every 3–4 generations.

### Candida albicans.

*C*. *albicans* strain SC5314 was used for all experiments, unless otherwise noted. GFP tagged *C*. *albicans* (gift from Robert T. Wheeler; Department of Molecular & Biomedical Sciences, University of Maine) (32) was used for the phagocytosis assays. Both strains of *C*. *albicans* were propagated on Sabouraud Glucose Agar (SGA) plates containing chloramphenicol (MilliporeSigma). Single colonies were subcultured overnight in liquid yeast peptone dextrose (YPD) media (MilliporeSigma) at 30°C in a shaking incubator. Culture (1 mL) was washed 3 times in PBS (Wisent); then, fungal cells counted by hemocytometer and the sole presence of yeast cells were confirmed by bright-field microscopy. Washed *C*. *albicans* yeast were diluted to the appropriate concentration per experiment in BMDM media. Dilutions were confirmed by quantitative culture on SGA plates incubated at 30°C for 48 hours.

### I.v. injection.

Washed and diluted *C*. *albicans* yeast from overnight culture were prepared in PBS at the concentration indicated for each experiment. Mice were heated under a red lamp for 4 minutes per cage, then 100 μL per mouse of *C*. *albicans* in PBS, or PBS alone, was injected via lateral tail vein. Mice were monitored immediately after injection until normal grooming resumed.

### Fungal burden.

At each time point, p.i. mice were euthanized by treatment with isoflurane followed by CO_2_ according to the ethical guidelines of the animal resource department of the McGill University Health Centre. Tissues were aseptically removed, weighed, and placed in 1 mL PBS on ice. All tissue was mechanically homogenized and then serially diluted in PBS. Dilutions were plated in duplicate on Sabouraud agar plates containing chloramphenicol and incubated at 30°C for 48 hours, and CFU was enumerated. CFU below detection (zero CFU) are listed as “1” when graphed in log-scale.

### Histopathology.

Mice were perfused with 20 mL of 10% formalin by intracardiac puncture. Tissues were stored in 10% formalin before being embedded in parafilm, sectioned, and stained with Periodic acid–Schiff (PAS) stain, H&E stain, or GMS stain. Images were obtained by Aperio AT Turbo digital slide scanner using a 20× objective and close-ups with a bright-field microscope using a 40× objective. p.Y91H patient biopsies were either stained with H&E or IHC was performed with neutrophil elastase antibody (Abcam, clone ERP7479), dilution 1/1,000, with pretreatment with CC1 on a Ventana Discovery instrument. Human H&E-stained samples were imaged with 400× magnification using a BX53 Olympus microscope and DP21 Olympus digital camera.

### Survival and observation of mouse behavior.

Mice were weighed, and original weight was recorded. Then, mice were observed for baseline bright, alert, and responsive behavior. Mice were injected with *C*. *albicans* by lateral tail vein in the amount listed for each experiment. Mice were weighed daily and monitored for signs of morbidity including piloerection, hunching, squinting, and lethargy throughout the 30-day experiment. Endpoint criteria were loss of 20% original weight, or 3 or more signs of morbidity combined with lethargy. Mice that displayed symptoms such as shaking, head tilting, altered gait, or unidirectional walking but were alert and responsive without weight loss were monitored twice daily. Videos of mouse behavior and images of anatomy were taken with an iPhone SE 12-megapixel camera.

### BMDM.

Whole bone marrow from 2 femurs was aseptically isolated in 1 mL PBS. The single-cell suspension (10 μL) was treated with ammonium-chloride-potassium (ACK) lysis solution to lyse RBCs and washed in PBS. Live cells from this sample were identified by 4% trypan blue exclusion and counted by hemocytometer under a bright-field microscope. Eight million bone marrow cells were added to 10 cm sterile Petri dishes containing 10 mL of BMDM media and 100 ng/mL of M-CSF (Peprotech). After 3 days, 4 mL of BMDM media and 50 ng/mL of M-CSF were added to cultures. BMDM were seeded for experiments after 6 days in culture. BMDM media were RPMI-1640 (Wisent) supplemented with 10% FBS (Wisent), 10 mM HEPES (Wisent), 1 mM sodium pyruvate (Wisent), 1% essential and nonessential amino acids (Wisent), and 100 U/mL penicillin/streptomycin (Wisent) and pH balanced with 5N NaOH.

### BMDM in vitro infection.

BMDM from mice of each genotype were seeded into 6-well plates at 1 million cells per well and allowed to adhere overnight (16–20 hours). *C*. *albicans* were prepared as for in vivo infection. BMDM were challenged with live *C*. *albicans* at an multiplicity of infection (MOI) of 1 in BMDM media for 2 hours followed by RNA extraction. BMDM were pulsed with *C*. *albicans* but immediately washed with PBS, and RNA extracted were used as corresponding sham-treatment controls.

### RT-PCR.

RNA from BMDM was extracted using TRIzol Reagent (Invitrogen) according to manufacturer’s instructions. In total, 500 ng of RNA was reverse-transcribed using the Maxima H Minus First Strand cDNA Synthesis Kit (Thermo Fisher Scientific). PCR for *Card9* cDNA was done using Phusion High-Fidelity DNA Polymerase (Thermo Fisher Scientific) according to the manufacturer’s instructions.

### qPCR.

TaqMan Gene Expression Assays were conducted on an Applied Biosystems 7300 Real-Time PCR System using TaqMan Fast Advanced Master Mix (Applied Biosystems, 4444554) according to manufacturer’s instructions for detection of *Il6* (Mm00446190_m1), *Il1b* (Mm01336189_m1), *Csf2* (Mm00438328_m1), and *Tnfa* (Mm00443258_m1). Targeted genes were normalized with reference genes *B2m* (Mm00437762_m1) and beta-actin (Mm02619580_g1). The differences between samples were calculated using the ΔΔCT method. Fold-change is reported relative to levels observed in sham-treated BMDM. BMDM derived from 12 mice per genotype were used.

### BMDM phagocytosis assay.

After 6 days in culture, BMDM were seeded into 24-well plates containing a round micro cover glass (Electron Microscopy Sciences, 72230-01) in each well, at 0.3 million cells per well and allowed to adhere overnight (16–20 hours). GFP-tagged *C*. *albicans* were prepared as for in vivo infection. BMDM were challenged with live *C*. *albicans* at an MOI of 1 and 0.1 for 15, 30, and 60 minutes and then fixed with 2% paraformaldehyde (PFA) in PBS for 10 minutes (Thermo Fisher Scientific, J19943-K2). To distinguish between intracellular and extracellular *C*. *albicans*, extracellular yeast were differentially stained with Calcofluor White (Sigma-Aldrich, 18909-100ml-F) for 10 minutes and washed with PBS. BMDM pulsed with *C*. *albicans* but immediately fixed with 2% PFA were used as 0-minute baseline controls. Samples were air dried and mounted on precleaned microscope slides (Thermo Fisher Scientific, 12-550-15) using IMM mounting medium (Ibidi, 50001). Imaging was done with an Echo 4-in-1 fluorescence microscope (Discover Echo Inc., RVL2-K2). Images were obtained using a 20× objective lens, and a minimum of 5 random fields were analyzed for each sample. Cell counting was performed manually with open resource Fiji software (ImageJ 1.53t). Percent Phagocytosis was calculated as follows: % Phagocytosis = PI. PI was calculated as follows: PI = (total number of engulfed *C*. *albicans* / total number of counted BMDM) × (number of BMDM containing engulfed *C*. *albicans* / total number of counted BMDM) × 100. Data from 9 mice per genotype were pooled for analysis.

### XTT assay.

BMDM were plated at 9 × 10^5^ cells per well in 12-well plates. The next day, cells were infected with 9 × 10^4^ live *C*. *albicans* (MOI, 0.1), centrifuged 3 minutes at 800*g*, and incubated at 37°C for 6 hours. BMDM lysis was achieved using a 0.1% Triton X-100 solution, and the remaining yeast was incubated in PBS containing 500 μg/mL XTT (X6493, Invitrogen) and 50 μg/mL Coenzyme Q0 (20504, Cayman Chemical) for 2 hours at 37°C. DMSO was then added to each well before recuperating and centrifuging the solution at 15,493*g* for 5 minutes. Absorbance was measured at 450 nm and 650 nm using a microplate reader, and the net absorbance (450–650 nm) was used for analysis.

### Western blot.

Protein from BMDM was isolated using RIPA buffer (Thermo Fisher Scientific) containing cOmplete Mini Protease Inhibitor Cocktail (Roche) and PhosSTOP (Roche), before being boiled in Blot Sample Reducing Agent (Thermo Fisher Scientific) and sample buffer (Novex). Protein was separated by SDS-PAGE on premade gels (Novex) before transfer to membrane via iBlot Gel Transfer Device (Invitrogen). Membrane was blocked in 10% skim milk in TBST, before being blotted using rabbit anti-CARD9 polyclonal antibody (Abnova, PAB12874) and mouse anti-GAPDH antibody (MilliporeSigma, MAB374) overnight at 4°C shaking. Membrane was washed in TBST and then blotted with secondary antibodies: goat anti–rabbit IgG DyLight 800 (Invitrogen, SA5-35571) and goat anti–mouse IgG DyLight 680 (Invitrogen, 35518) for 1 hour at room temperature, with gentle agitation. Membrane was washed in TBST and then with PBS before being imaged on an Odyssey Infrared Imager (v3.0, LI-COR) using Odyssey Application Software (LI-COR).

### Sanger sequencing.

The exon 3 of the CARD9 gene was PCR amplified from genomic DNA using primers designed to flank c.T271. Sequencing was performed at the McGill University and Génome Québec Innovation Centre. Sequencing analyses were performed on Sequencher sequence analysis software (Gene Codes Corporation).

### CT-scan of mouse skull.

The scan was done with a nanoScan SPECT/CT (Mediso USA), at 35 kVp x-ray source voltage 980 μA current, and 450 ms exposure time, and the scan method was semicircular. The tomographic reconstruction of the projections resulted in 1,075 slices with isotropic 20 μm voxel size.

### Single-cell suspensions.

For brain, spleen, and kidney, mice were ethically euthanized before being perfused with 30 mL PBS by intracardiac puncture. Whole tissue was aseptically harvested, weighed, minced in minimal PBS, and was then placed in 1× HBSS (Wisent) containing collagenase D (MilliporeSigma) and DNase I (MilliporeSigma) on ice. Tissue was digested at 37°C for 30 minutes in a shaking incubator (200 rpm); it was then passed through a 100 μm cell strainer into a total volume of 17 mL 1× HBSS. After centrifugation at 300*g*, cells were brought up in 10 mL of 37% Percoll (GE Healthcare) in HBSS, spun at 500*g* for 10 minutes, and allowed to come to a stop with no brake. Myelin (brain) and debris (kidney, spleen) was removed by vacuum, and cells were washed in 5 mL 1× HBSS. After RBC lysis in ACK, single-cell suspensions were resuspended in 200 μL FACS buffer (0.5% BSA in PBS, MilliporeSigma) for enumeration. Live cells were identified by 4% trypan blue exclusion and counted by hemocytometer under a bright-field microscope. For bone marrow, both femurs were aseptically removed, and the ends cut to isolate the femoral shaft. Bone marrow was isolated by centrifugation at 2,000*g* into 200 μL RPMI media, the volume of both femurs combined, and the total volume increased to 1 mL RPMI. Live cells were identified by 4% trypan blue exclusion and counted by hemocytometer under a bright-field microscope. In total, 1 million cells per mouse were then treated with ACK for RBC lysis and stained for flow cytometry.

Blood was collected by cardiac puncture into heparin coated tubes (BD Microtainer tubes with lithium heparin). In total, 25 μL of blood was directly stained for flow cytometry before being brought up in ACK for RBC lysis and washed with FACS buffer. Stained blood samples were then fixed in 1% PFA.

### Flow cytometry.

Single-cell suspensions were stained with viability dye eFluor 506 (Invitrogen, 50-246-097; 20 minutes; 4°C) and surface blocked with anti-CD16/32 (BD Biosciences, clone 93) in 0.5% BSA/PBS solution to block nonspecific antibody interaction with Fc receptors (10 minutes; 4°C). Cells were then surface stained with anti-CD45.2 in BV421 (eBioscience, clone 104), anti-CD11b in PE-Cy7 (BD Bioscience, clone M1/7), anti-Ly6G in Alexa Fluor 700 (BioLegend, clone 1A8), anti-Ly6C in APC (BioLegend, clone AL-21), anti-CD11c in PerCP Cy5.5 (eBioscience, clone N418), anti-CX3CR1 in BV650 (BioLegend, clone SA011F11), anti-CD3 in PE (BioLegend, clone 145-2C11), anti-CD19 in BV786 (BD Biosciences, clone 1D3), anti-MHCII in BV605 (BD Biosciences, clone M5/114.15.2), anti-NK1.1 in APC-780 (Invitrogen, clone PK136), anti-SiglecF in PE-CF594 (BD Horizon, clone E50-2440), and anti-CD64 in FITC (BioLegend, clone X54-5/7.1). Controls were stained with FMO antibody cocktails. Samples and controls were both stained for 30 minutes at 4°C. Cells were washed once in FACS buffer and then fixed in 1% PFA (Alfa Aesar) in PBS. Flow cytometry was performed using LSRFortessa X-20 (BD Biosciences) with FACSDiva Software version 8.0.1 (BD Biosciences). Analysis was performed using FlowJo software version 10.4.2.

### Bone marrow–derived neutrophils.

Whole bone marrow was flushed from 2 femurs and 2 tibias using RPMI 1640 (Wisent) supplemented with 10% FBS (Wisent) and 2 mM EDTA (Wisent). Cell suspension was filtered on a 100 mm filter, and cells were counted by hemocytometer under a bright-field microscope. Neutrophils were purified by negative magnetic bead selection using MojoSort Mouse Neutrophil Isolation Kit (BioLegend) according to the manufacturer’s instructions. Bone marrow–enriched neutrophils (CD45^+^CD11b^+^Ly-6G^+^) had > 80% purity and > 90% viability as determined by flow cytometry using FACSCanto II cytometer (BD Biosciences).

### Neutrophil killing assay.

Neutrophil killing of *Candida albicans* was determined by CFU enumeration. Bone marrow–enriched neutrophils were seeded into 48-well plates at 0.25 million cells per well and allowed to settle for 30 minutes at 37°C, 5% CO_2_, while GFP tagged *C*. *albicans* was opsonized with 10% fresh mouse serum in 1× HBSS for 30 minutes at 4°C. Unopsonized or serum-opsonized *C*. *albicans* were added to neutrophils at an MOI of 0.05 for 3 hours; then, cells were lysed with 0.02% Triton X-100 in ice-cold water for 5 minutes and plated. Neutrophil killing was calculated as follows: (CFU from wells with neutrophils) / (CFU from control wells without neutrophils) × 100%.

### Neutrophil phagocytosis assay.

In total, 0.5 million bone marrow–enriched neutrophils were incubated with unopsonized or mouse serum–opsonized GFP tagged *C*. *albicans* at an MOI of 0.5 in a 2 mL round-bottom tube for 30 minutes on a revolver rotator end-to-tail at 37°C, 5% CO_2_. Infected neutrophils were then stained with 1:500 anti–Ly-6G in APC (BioLegend, clone 1A8) in PBS with 1:100 anti-CD16/CD32 Fc block (BD Biosciences, clone 93) for 30 minutes at 4°C. To distinguish between intracellular and extracellular *C*. *albicans*, extracellular yeasts were differentially stained with Uvitex 2B (Polysciences) in PBS for 1 minute at RT and washed with PBS then fixed with 1% PFA (Alfa Aesar). Samples were analyzed on the Amnis ImageStream^X^ Mk II imaging flow cytometer with INSPIRE software (Cytek Biosciences) at the RI-MUHC Immunophenotyping core platform. Data analysis was done using the IDEAS v6.2 software (Cytek Biosciences). The neutrophils associated with *C*. *albicans* (this population includes neutrophils with both internalized and neutrophil-bound yeasts) is shown as a percentage of GFP^+^Ly-6G^+^ neutrophils. Percent of neutrophils with internalized *C*. *albicans* is the percentage of gated Uvitex^–^GFP^+^Ly-6G^+^ neutrophils. The number of yeasts per neutrophil was calculated using the Spot Count Wizard to create a GFP spot count feature in the Uvitex^–^GFP^+^Ly-6G^+^ gate.

### Clodronate cell depletion.

Mice were injected i.p. with 200 mL of clodronate or control liposomes (Encapsula Nano Sciences) 24 hours and 1 hour prior to *C*. *albicans* i.v. infection, as well as 24 hours and 72 hours after infection (4 injections total). At day 4 after infection, kidneys and brain were aseptically removed, and fungal burden was assessed as described above. For analysis of the blood by flow cytometry, blood was collected by cardiac puncture into heparin-coated tubes (BD Microtainer tubes with lithium heparin). In total, 50 μL of blood was directly surface blocked with anti-CD16/32 (BD Biosciences, clone 93) and stained with anti-CD45.2 in BV421 (eBioscience, clone 104), anti-CD11b in PE (eBioscience, clone M1/7), anti-Ly6G in APC (BioLegend, clone A18), and anti-CD115 in PerCP-eF710 (eBioscience, clone AFS98), for 30 minutes at 4°C. RBC lysis was performed with FACS Lysis Solution (BD Biosciences) for 10 minutes at room temperature. Cells were washed once in FACS buffer and then fixed in 1% PFA (Alfa Aesar) in PBS. Flow cytometry was performed using FACSCanto II cytometer (BD Biosciences) with FACSDiva Software version 8.0.1 (BD Biosciences). Analysis was performed using FlowJo software version 10.4.2.

### Primers.

Primers are listed in the 5′ to 3′ direction and are as follows: Card9 exon 3 amplification forward primer (Fwd): GCAGGGCGCCTTATTCAATG, reverse primer (Rev): GGCTCCCCTTCTAGAGACCA; c.T271C sequencing primers Fwd: CATCTCCAAGAGCCTCCACC, Rev: TCATAGAAGCCAGGACCCGA; and Card9 cDNA (RT-PCR) Fwd: CTCACTGCCTCAGGATCTGG, Rev: CCCTGTCTGCCAGTACACCT. For Card9^–/–^ genotyping (The Jackson Laboratory) the common Fwd is CTGACAGGGAACAGAAGGTG; the Rev WT is AGGACTTTGCACTGGCGTAG; and the Rev KO is TGCCTGCTTGCCGAATATC.

### Statistics.

Statistical analysis was performed using GraphPad Prism 8.0.2 software. Unless otherwise specified, 1-way ANOVA followed by a multiple-comparison test (Tukey’s) was used to test differences between groups. A log-rank test was used for Kaplan-Meier survival curves to test for significance. Kruskal-Wallis was used for nonparametric data (qPCR and phagocytosis assay), and the Mann-Whitney *U* test was used to compare 2 nonparametric samples (clodronate data). A 2-way ANOVA was used for grouped data, followed by a multiple comparisons test (Holm-Šídák). For paired data, multiple 2-tailed t tests were performed. *P* < 0.05 was considered significant.

### Study approval.

The mouse study was conducted under the RI-MUHC–approved animal use protocol 7829 (Animal Care Committee of McGill University). The human study was reviewed and approved by the MUHC Research Ethics Board, Montréal, Québec, Canada (protocol no. GEN10-256). All patients provided informed consent prior to their participation in the study. All animals were maintained in compliance with the Canadian Council on Animal Care, and all experiments were approved by the McGill University animal care and use committee.

### Data availability.

Values for all data points in graphs can be found in the Supporting Data Values file.

## Author contributions

DCV, MD, and ML conceptualized the project. ML performed all animal infections and experiments as well as downstream analysis. ML created and assembled all figures. IA, A Beauchamp, and STQ generated the Y91H^KI^ mice and assisted with breeding. IA performed ex vivo neutrophil experiments and, together with A Beauchamp, performed the clodronate experiments. A Boisvert assisted with fungal burden experiments and animal harvests. MAD performed the XTT assay. YL performed the phagocytosis and qPCR experiments in BMDM. LR assisted with fungal burden experiments and provided helpful discussions. WBZ assisted with fungal burden experiments. CG assisted with animal harvests. JL helped prepare the figures. SB, MKB, YS, BS, MSL, and ILK provided helpful discussions and experimental support. MCG provided human histopathology data. RTW provided the *C*. *albicans* and technical support. ML, MD, and DCV drafted and edited the manuscript. DCV and MD supervised the project.

## Supplementary Material

Supplemental data

Unedited blot and gel images

Supplemental video 1

Supplemental video 2

Supporting data values

## Version 1 05/27/2025

In-Press Preview

## Version 2 07/08/2025

Electronic publication
