# Supplementary material for: A CARD9 deficiency mouse model recapitulates human chronic CNS candidiasis identifying defective monocytic cell responses in immunopathogenesis
Source: JCI Insight. 2025 May 27;10(13):e176676. doi: 10.1172/jci.insight.176676 (PMC12288897; doi:10.1172/jci.insight.176676)
Supplement: Supplemental data [file jciinsight-10-176676-s134.pdf]

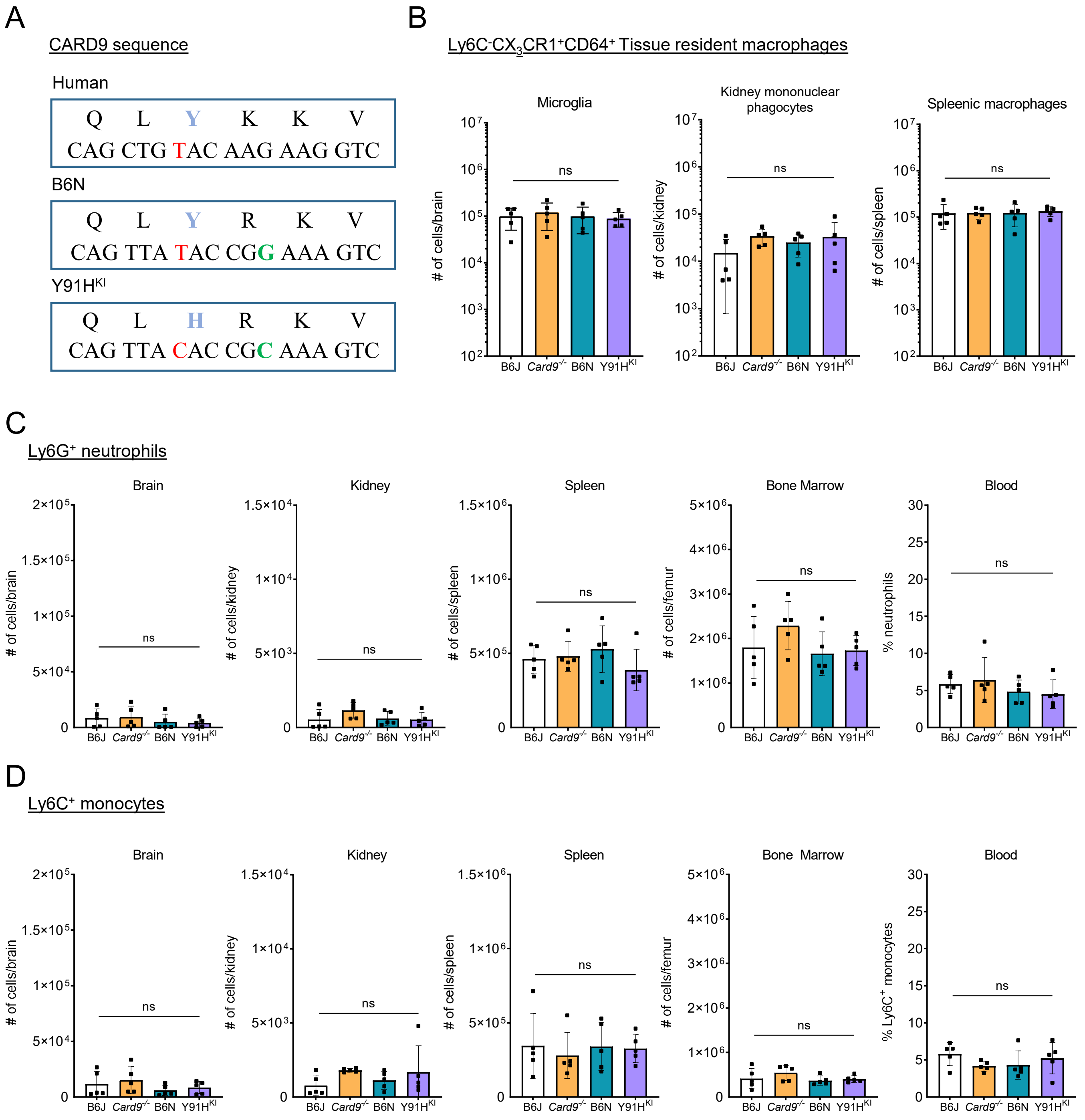

Supplementary Figure 1. CARD9 p.Y91 is conserved between mice and humans and CARD9-deficient mice have no steady state defects in myeloid cell numbers. **A)** Schematic representation showing the genomic and predicted amino acid sequences of human (top), C57BL6/N mice (B6N, middle) and Y91H^{KI} mice (bottom). Blue indicates the conserved tyrosine in human and B6N, and the mutated histidine in the Y91H^{KI} amino acid sequences. Red indicates the conserved c.T271 in human and B6N, and the mutated c.T271C in the Y91H^{KI} genomic sequence. The PAM site is indicated in green. **B)** Flow cytometric analysis of tissue resident macrophage populations shown, identified as live CD45^{+}CD11b^{+}Ly6G^{-}Ly6C^{-}CX$_3$CR1^{+}CD64^{+} cells. **C)** Flow cytometric analysis of neutrophil populations in the tissues indicated. Neutrophils were identified as live CD45^{+}CD11b^{+}Ly6G^{+} cells. **D)** Flow cytometric analysis of monocyte populations in the tissues indicated. Monocytes were identified as live CD45^{+}CD11b^{+}Ly6G^{-}Ly6C^{+} cells. Means +/- SD shown. n=5 mice per group, 2 experiments pooled. Kruskal-Wallis test for significance performed. ns: not significant.

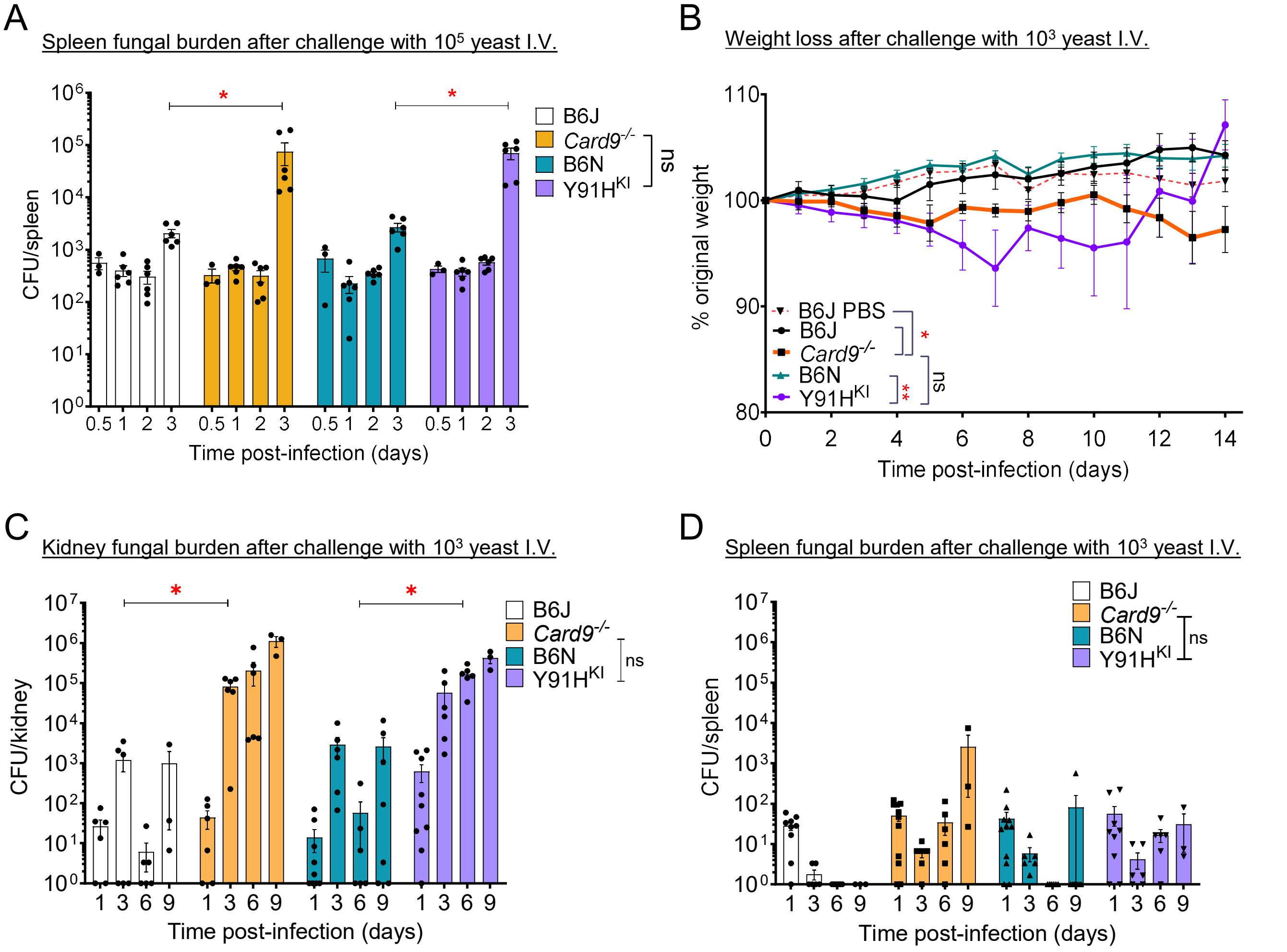

Supplementary Figure 2. Disseminated disease and morbidity characterizing *C. albicans* infection in CARD9-deficient mice. A) Dot plots show tissue fungal burden from mice injected via lateral tail vein with 1×10^5 *C. albicans* yeast cells. n=3-6 mice, 2 experiments pooled. Mean +/- SEM shown. Two-way ANOVA with Tukey's multiple comparisons test. **P<0.01, ***P<0.001, ns: not significant. **B)** Weight loss of mice injected via lateral tail vein with 1×10^3 CFU of *C. albicans* or PBS alone (B6J PBS). Mean +/- SEM shown. Friedman test with Dunn's multiple comparisons test. *P<0.05, **P<0.01, ns: not significant. B6J PBS: n=8; B6J: n=11; *Card9*$^{-/-}$: n=14; B6N: n=15; Y91H^{KI}: n=12. 3 pooled experiments. **C&D)** Dot plots show tissue fungal burden in the kidney **(C)** and spleen **(D)** from mice injected via lateral tail vein with 1×10^3 *C. albicans* yeast cells. n=3-8 mice, 2 experiments pooled. The means +/- SEM are shown. Two-way ANOVA with Tukey's multiple comparisons test. *P<0.05, ns: not significant.

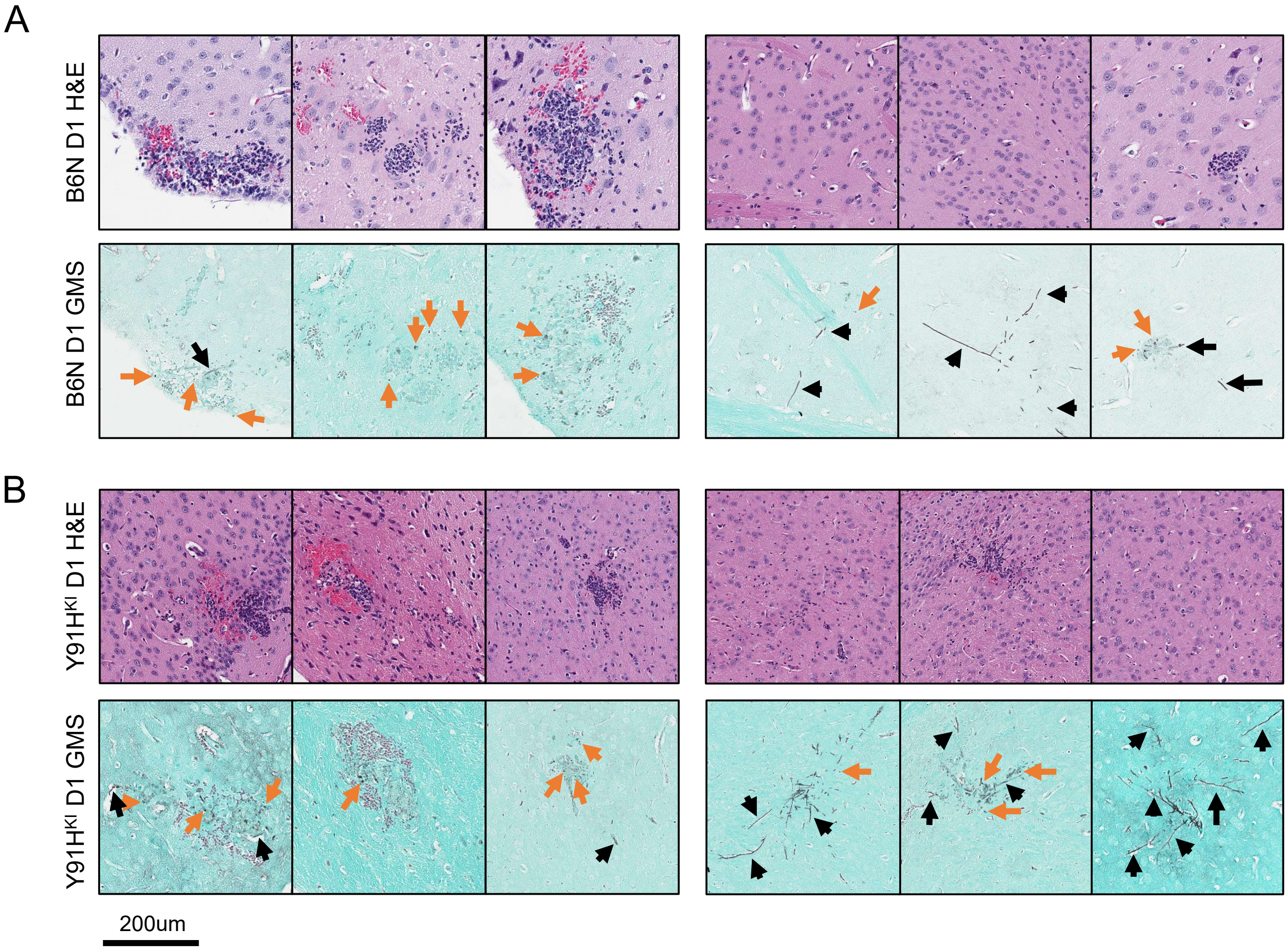

Supplementary Figure 3. Detailed analysis of early fungal lesions by histopathology staining. Six lesions per genotype (3 images per mouse) shown as representative images from 2 experiments. Mice at day 1 (D1) post-infection with $1x10^3$ *C. albicans* yeast cells. GMS and H&E staining were done on serial sections and images shown are matched for anatomical location. **A)** Wild-type B6N mice. **B)** Y91H^{KI} mice. Orange arrows indicate *C. albicans* yeast cells; black arrows indicate hyphae. Images taken with a 20x objective. Scale bar shows 200microns for all images.

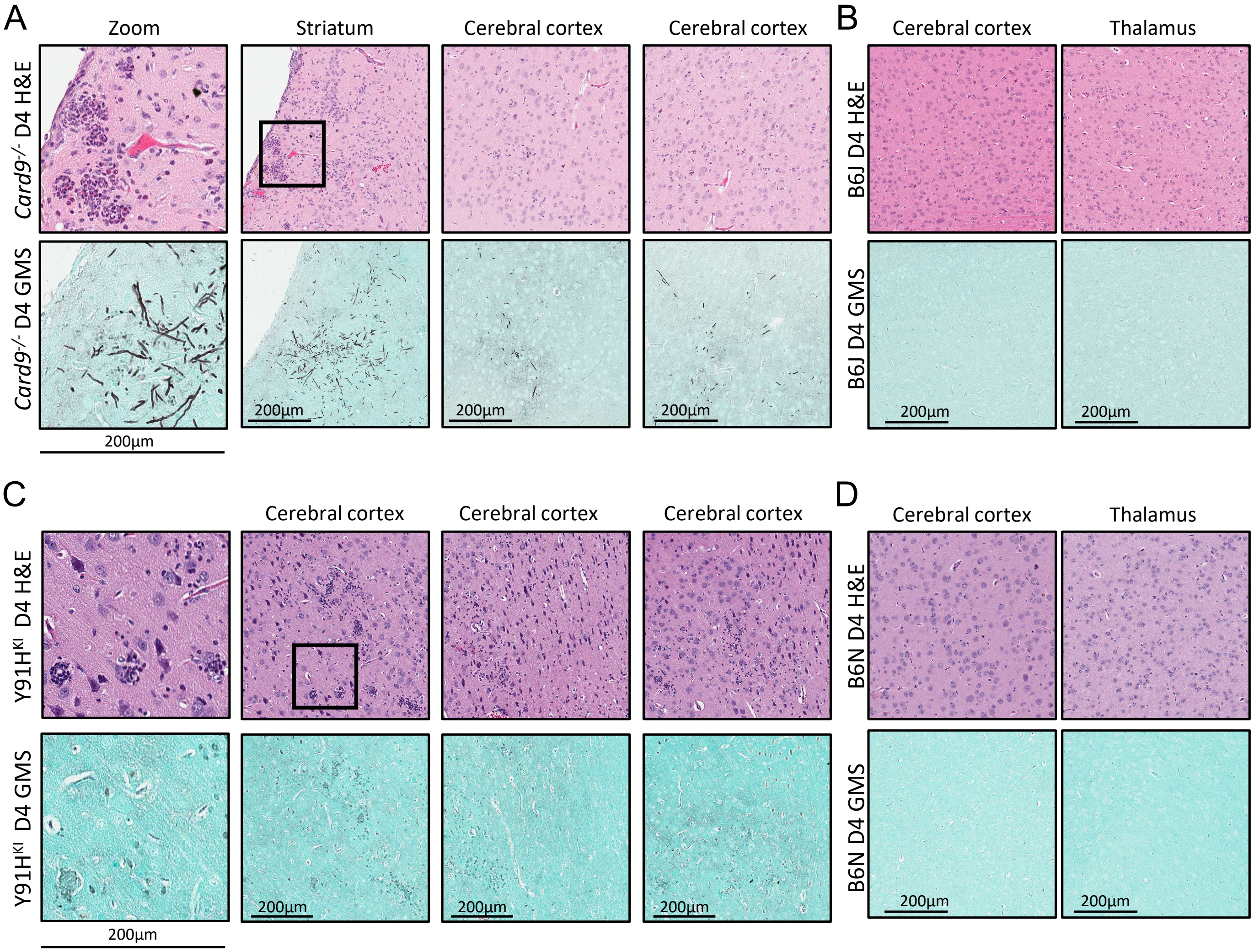

Supplementary Figure 4. Detailed view of fungal lesions and inflammation in CARD9-deficient mouse brain by histopathology staining at day 4 post-infection. Histopathology staining of the brain of CARD9-deficient **(A, C)** and wild-type **(B, D)** mice at day 4 (D4) post-infection with $1x10^3$ *C. albicans* yeast cells. GMS and H&E staining were done on serial sections and images shown are matched for anatomical location. Representative images of the cerebral cortex and thalamus are shown for each genotype, as well as a representative image of severe lesions in CARD9-deficient mice. Black boxes indicate the location of zoomed images. Images were obtained by bright field microscopy with a 20x objective and are representative of 2 experiments.

A

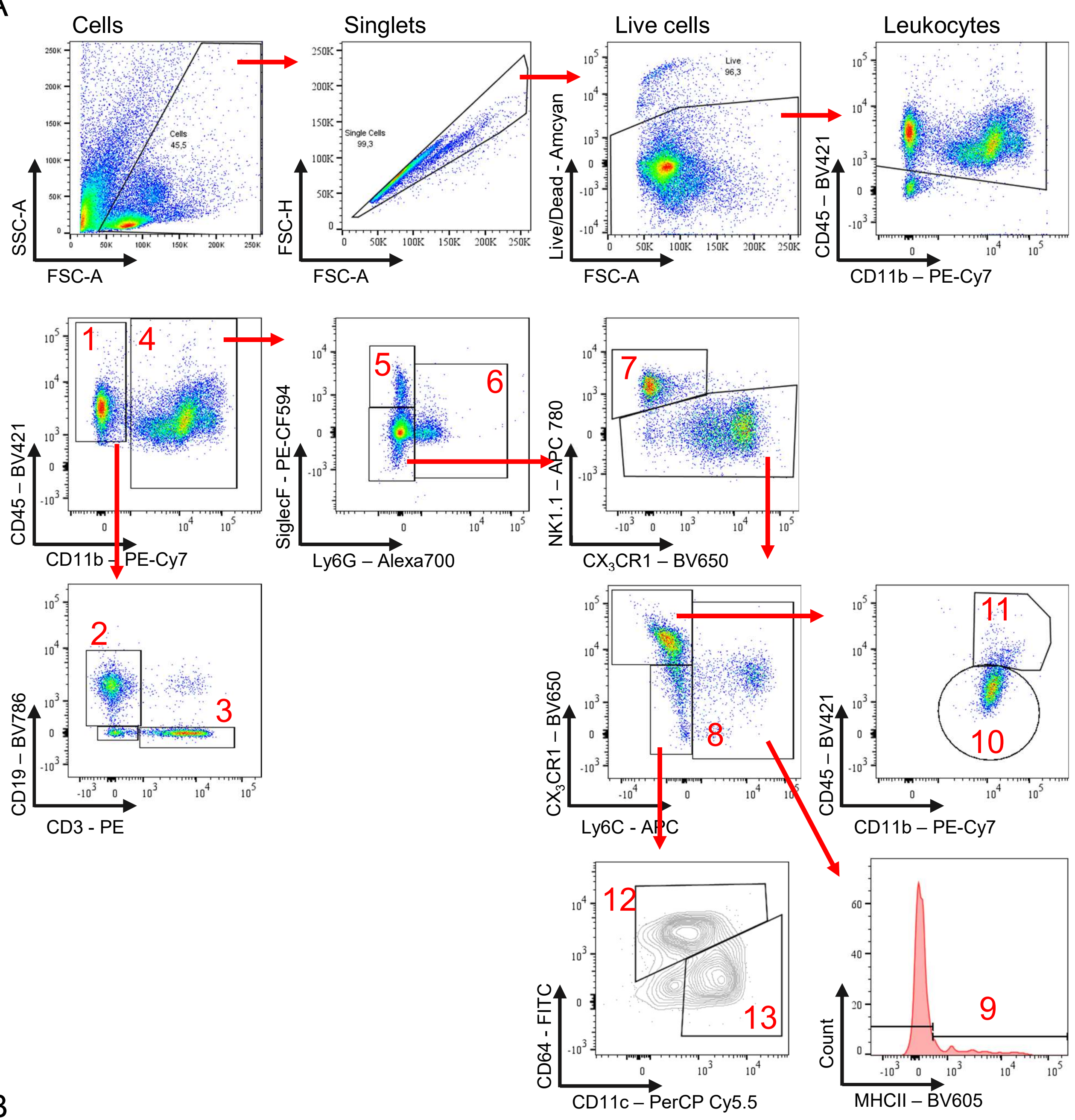

B

Surface markers defining immune cell populations				
	**Cell Type**	**CD45 & CD11b**	**Positive markers**	**Negative markers**
	Leukocytes	CD45$^+$		Live-dead stain$^-$
1	Lymphocytes	CD45$^+$CD11b$^-$		
2	B cells	CD45$^+$CD11b$^-$	CD19$^+$	CD3$^-$
3	T cells	CD45$^+$CD11b$^-$	CD3$^+$	
4	Myeloid cells	CD45$^+$CD11b$^+$		
5	Eosinophils	CD45$^+$CD11b$^+$	SiglecF$^+$	Ly6G$^-$
6	Neutrophils	CD45$^+$CD11b$^+$	Ly6G$^+$	
7	NK Cells	CD45$^+$CD11b$^+$	NK1.1$^+$	Ly6G$^-$SiglecF$^-$
8	Ly6C$^+$ Monocytes	CD45$^+$CD11b$^+$	Ly6C$^+$	Ly6G$^-$SiglecF$^-$NK1.1$^-$
9	MHCII$^+$Ly6C$^+$ Monocytes	CD45$^+$CD11b$^+$	MHCII$^+$Ly6C$^+$	Ly6G$^-$SiglecF$^-$NK1.1$^-$
10	Microglia	CD45^{int}CD11b^{int}	CX$_3$CR1^{hi}	Ly6G$^-$SiglecF$^-$NK1.1$^-$Ly6C$^-$
11	Ly6C$^-$ Monocytes	CD45^{hi}CD11b^{hi}	CX$_3$CR1^{hi}	Ly6G$^-$SiglecF$^-$NK1.1$^-$Ly6C$^-$
12	Macrophages	CD45$^+$CD11b$^+$	CD64$^+$	Ly6G$^-$SiglecF$^-$NK1.1$^-$Ly6C$^-$CX$_3$CR1$^{-/lo}$
13	cDC	CD45$^+$CD11b$^+$	CD11c$^+$	Ly6G$^-$SiglecF$^-$NK1.1$^-$Ly6C$^-$CX$_3$CR1$^{-/lo}$CD64$^-$

Supplementary Figure 5. Flow cytometry gating strategy for brain immunophenotyping. A) Flow cytometry plots showing the gating strategy for immunophenotyping in the brain. Numbers in red correspond to the cell types listed in B. **B)** Table showing the surface markers used to define each cell population.

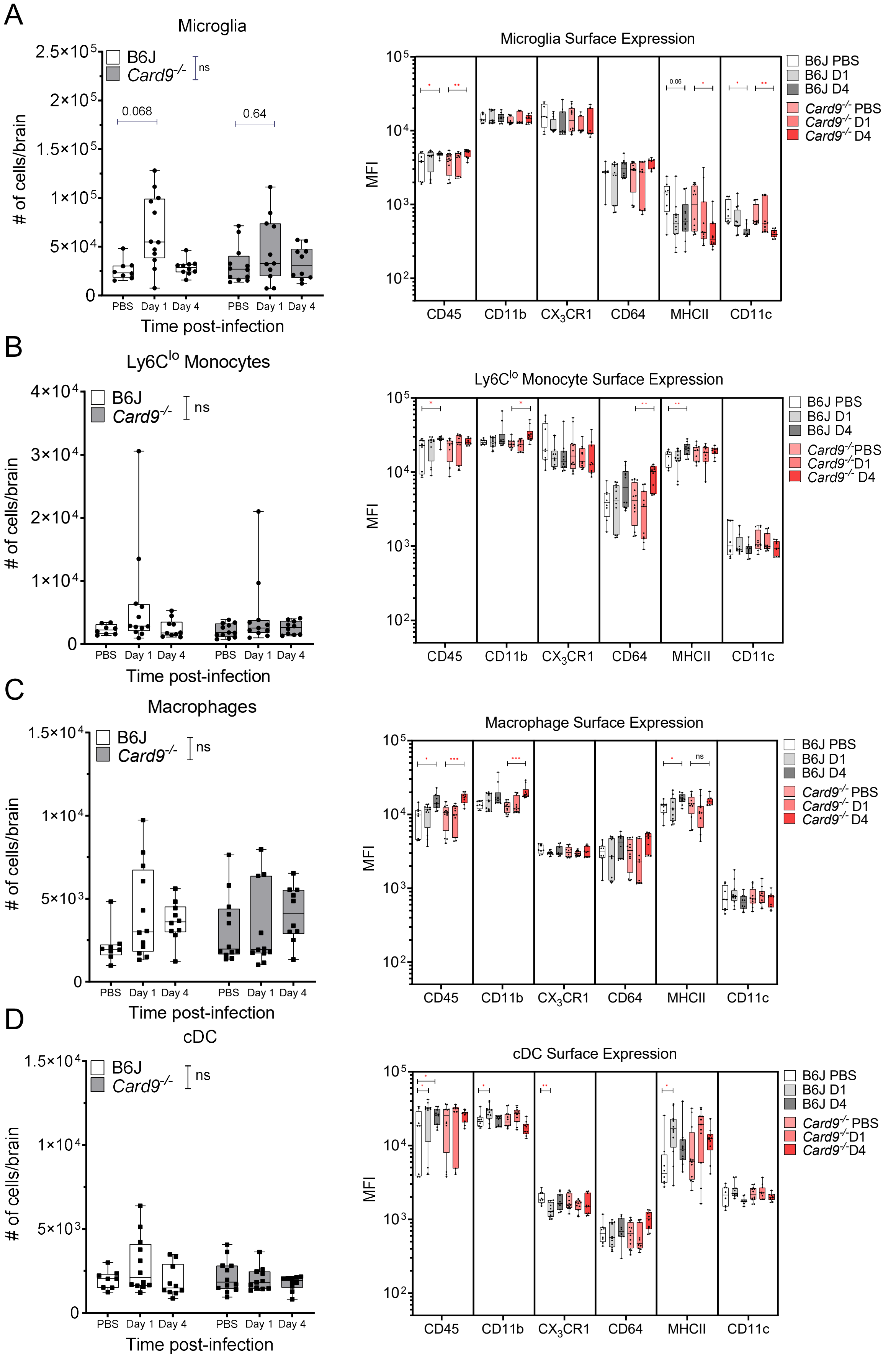

Supplementary Figure 6. Mononuclear phagocyte responses during chronic invasive candidiasis in *Card9*$^{-/-}$ mice. Box and whisker plots showing B6J and *Card9*$^{-/-}$ mice from figure 5 analyzed for mononuclear phagocyte numbers in the brain (left) and surface expression markers (right). **A)** Microglia ($CD45^{int}CD11b^{int}LIN^{-}CX_3CR1^{hi}$). **B)** Ly6C^{lo} Monocytes ($CD45^{hi}CD11b^{hi}LIN^{-}CX_3CR1^{hi}$). **C)** Macrophages ($CD45^{+}CD11b^{+}LIN^{-}Ly6C^{-}CD64^{+}$). **D)** cDC ($CD45^{+}CD11b^{+}LIN^{-}Ly6C^{-}CD64^{-}CD11c^{+}$). n=8-16 mice per group, 3 experiments pools. Median and interquartile range shown. Two-way ANOVA with Dunnett's multiple comparisons test for MFI. *P<0.05; **P<0.01; ***P<0.001. ns: not significant.

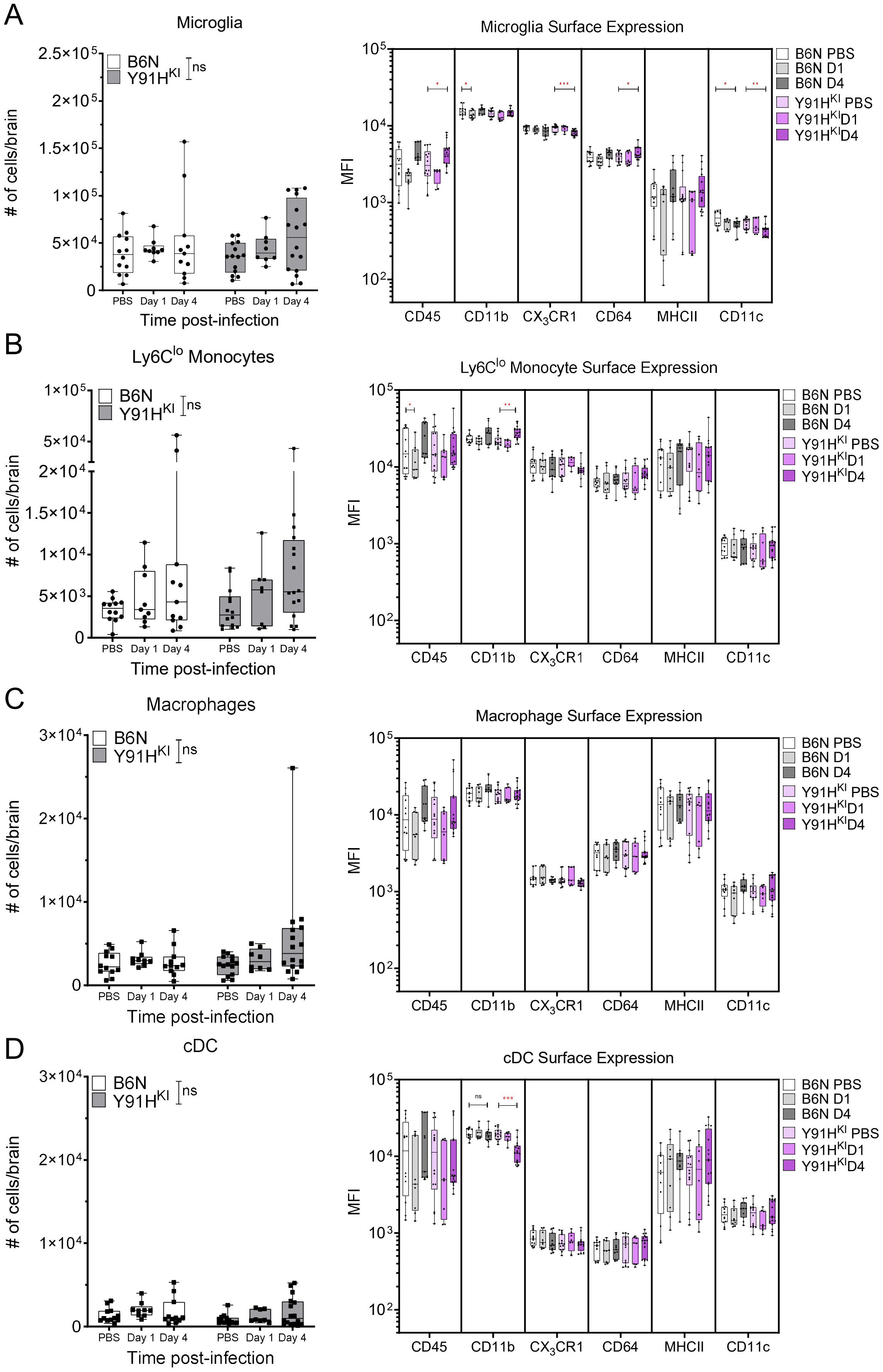

Supplementary Figure 7. Mononuclear phagocyte responses during chronic invasive candidiasis in Y91H^{KI} mice. Box and whisker plots showing B6N and Y91H^{KI} mice from figure 5 analyzed for mononuclear phagocyte numbers in the brain (left) and surface expression markers (right). **A)** Microglia (CD45^{int}CD11b^{int}LIN^{-}CX$_3$CR1^{hi}). **B)** Ly6C^{lo} Monocytes (CD45^{hi}CD11b^{hi}LIN^{-}CX$_3$CR1^{hi}). **C)** Macrophages (CD45^{+}CD11b^{+}LIN^{-}Ly6C^{-}CD64^{+}). D) cDC (CD45^{+}CD11b^{+}LIN^{-}Ly6C^{-}CD64^{-}CD11c^{+}). n=8-16 mice per group, 3 experiments pools. Median and interquartile range shown. Two-way ANOVA with Dunnett's multiple comparisons test for MFI. *P<0.05; **P<0.01; ***P<0.001. ns: not significant.

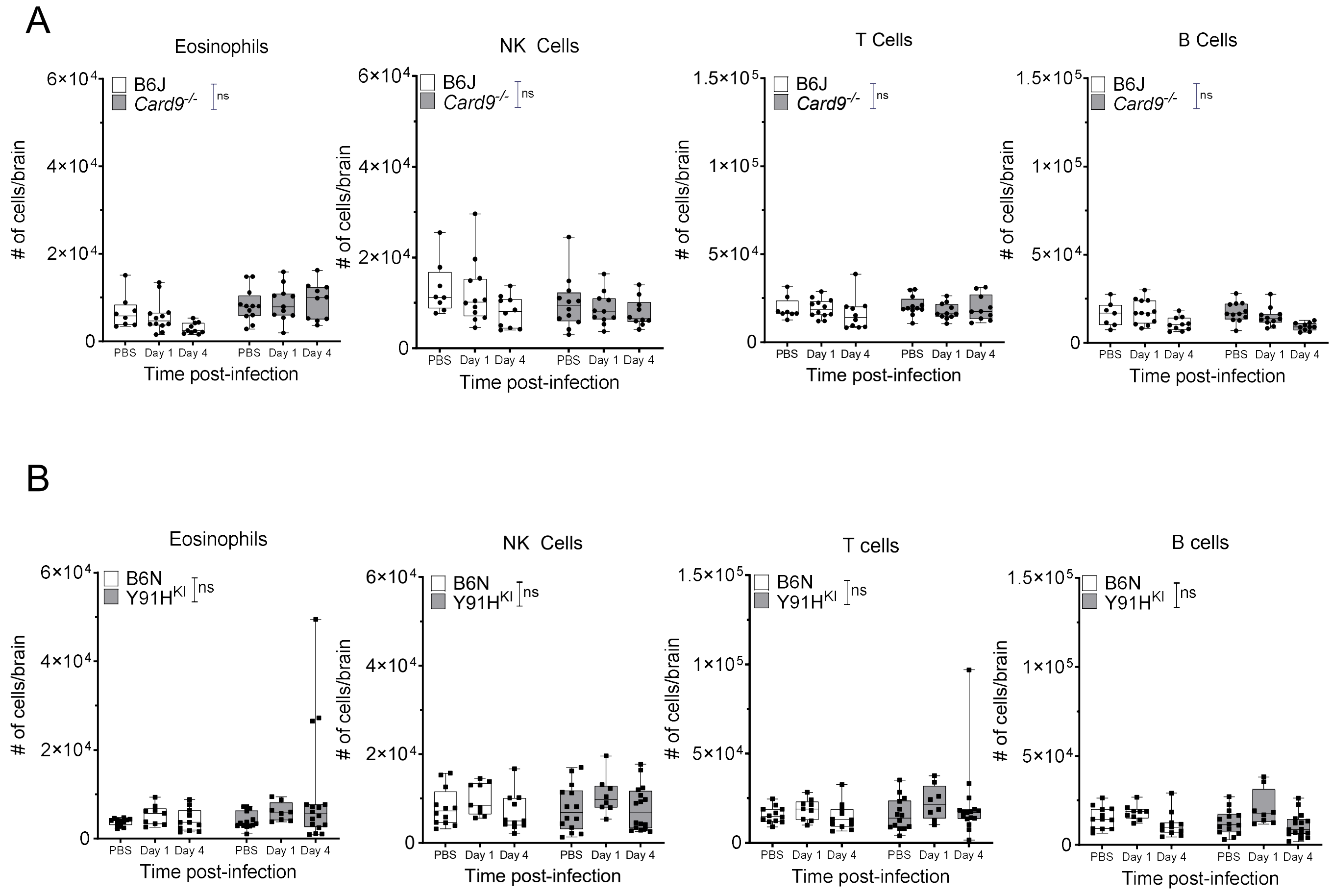

Supplementary Figure 8. Immunophenotyping of the brain during chronic invasive candidiasis in CARD9-deficiency. Box and whisker plots showing **A)** B6J and *Card9*$^{-/-}$ mice or **B)** B6N and Y91H^{KI} mice from figure 5 were analyzed for eosinophils ($CD45^+CD11b^+SiglecF^+Ly6G^-$), NK cells ($CD45^+CD11b^+LIN^-NK.1.1^+$), T cells ($CD45^+CD11b^-CD3^+$), and B cells ($CD45^+CD11b^-CD19^+$). n=8-16 mice per group, 3 experiments pools. Median and interquartile range shown. Two-way ANOVA with Sidak's multiple comparisons test. ns: not significant.

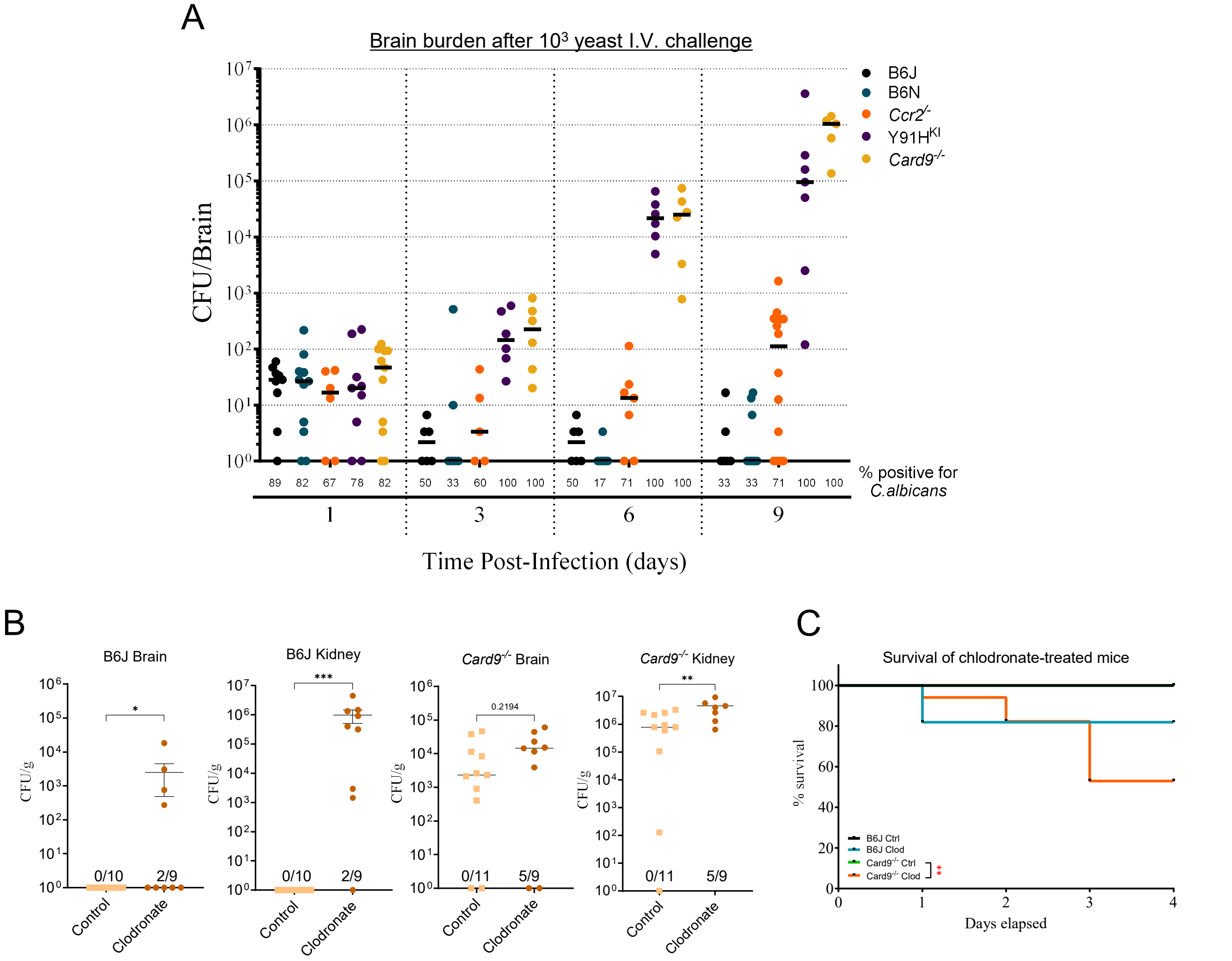

Supplementary Figure 9. Mononuclear phagocytes are protective in chronic invasive candidiasis and CARD9-deficiency. A) Brain fungal burden from mice injected via lateral tail vein with 1×10^3 *C. albicans* yeast cells. n=5-9 mice, 3 experiments pooled. Median is indicated with a black bar. Percentages list are (mice for each genotype with detectable brain fungal burden)/(total mice infected) x 100%. B6J,B6N, Y91H, and *Card9*$^{-/-}$ data are from Figure 3C. Data from *Ccr2*$^{-/-}$ mice was generated in the same experiments but excluded from Figure3C for brevity. **B)** Brain and kidney fungal burden on day 4 P.I. in B6J and *Card9*$^{-/-}$ mice. The mean +/- SEM are shown. Mann-Whitney test was performed. *P<0.05, **P<0.01, ***P<0.001. Ratio indicates the survivor bias of the data: # of mice that died/total mice infected. n=9-11mice, 3 experiments pooled **C)** Kaplan-Meier curve of mouse survival post-infection. B6N and Y91H mice had no deaths and are excluded for brevity. n=10-17mice, 3 experiments pooled. **P<0.01.

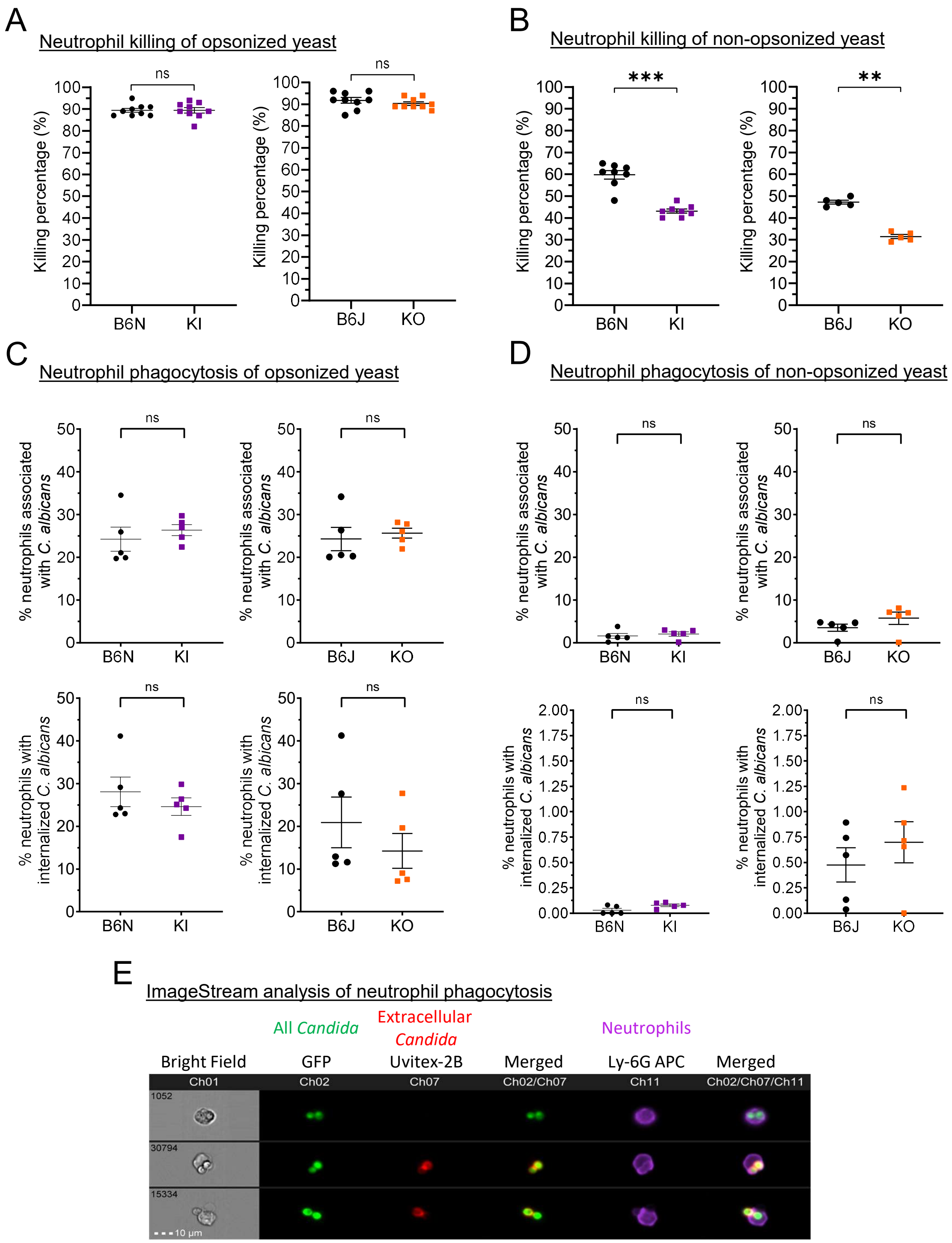

Supplementary Figure 10. CARD9-deficient neutrophils have impaired killing but not phagocytosis of yeast cells *ex vivo*. A) and B) Scatter plots show percent killing of *C. albicans* yeast after co-culture with *ex vivo* bone marrow neutrophils for 3hr at an MOI of 20:1. Percent killing was calculated as 1- (CFU after co-culture)/(CFU without neutrophils). **A)** Yeast were opsonized with fresh mouse serum for 30min, then added to the neutrophil culture. **B)** Yeast were added directly to the neutrophil culture without opsonization. n=5-9 mice, 5 experiments pooled. **C) and D)** Scatter plots show percent phagocytosis of *C. albicans* after 30min co-culture with *ex vivo* bone marrow neutrophils at an MOI of 2:1 and assessed by ImageStream flow cytometry. **C)** Yeast were opsonized with fresh mouse serum for 30min, then added to the neutrophil culture. **D)** Yeast were added directly to the neutrophil culture without opsonization. For all graphs, the mean +/- SEM are shown, n=5 mice, 5 experiments pooled. Mann-Whitney test was performed. **P<0.01, ***P<0.001, ns: not significant. **E)** Image shows an example of the differentiation between extracellular and intracellular *C. albicans* by ImageStream. KI: Y91H^{KI}. KO: *Card9*$^{-/-}$.

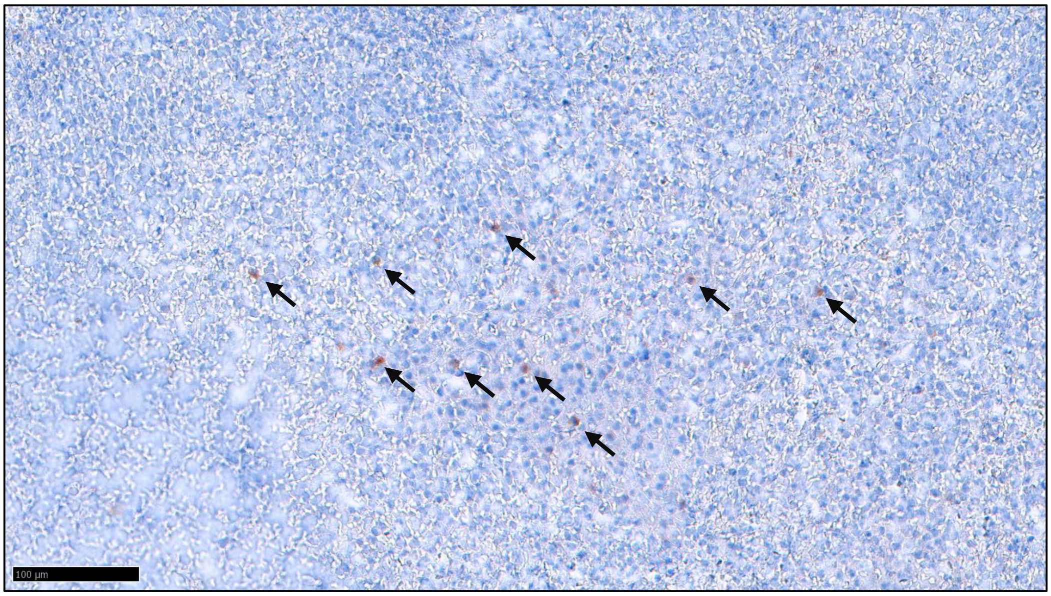

Supplementary Figure 11. CARD9-deficient p.Y91H patient brain biopsy confirms the presence of neutrophils. Immunohistochemistry of a human p.Y91H patient brain biopsy stained for neutrophil elastase. Microphotograph obtained at 200x magnification. Arrows highlight examples of positive neutrophil elastase staining. Scale is indicated at the bottom left, 100µm.

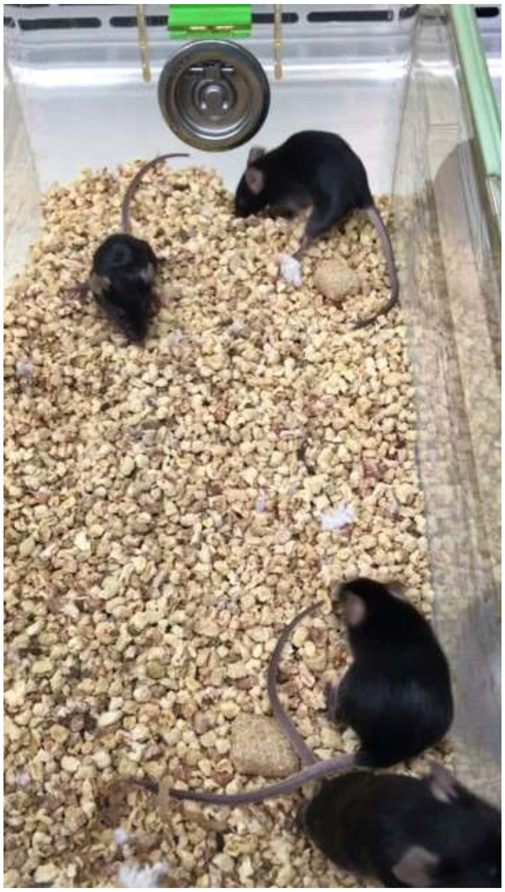

Supplementary Video 1. Y91H^{KI} mice display overt neurological symptoms during chronic invasive candidiasis. Video shows four Y91H^{KI} mice. Two mice are BAR (bright-eyed, active, and responsive) and therefore scored as asymptomatic. Mouse in the bottom right corner displays (clockwise) circling behaviour which was scored as "neurological symptom". Remaining mouse is small and displays altered gait, with poor motor control and balance, also scored as "neurological symptom".

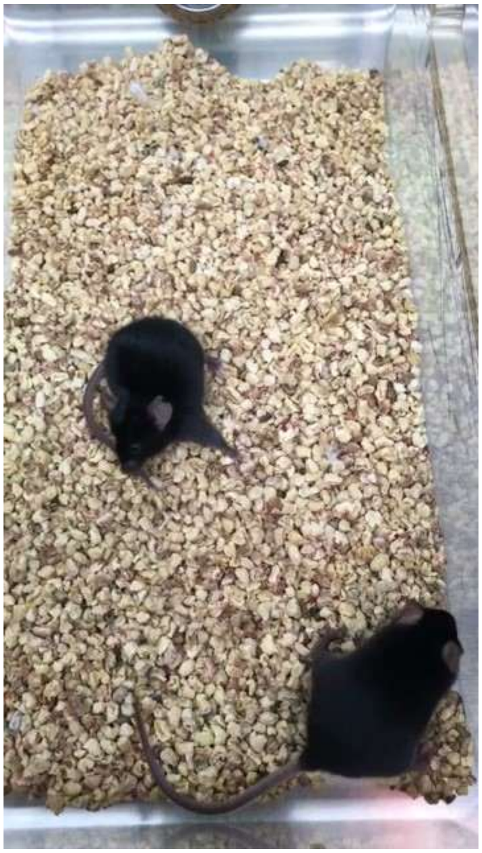

Supplementary Video 2. *Card9$^{-/-}$* mice display overt neurological symptoms and develop skull distention during chronic invasive candidiasis. Video shows two *Card9$^{-/-}$* mice 21 days post-infection. One mouse is BAR (bright-eyed, active, and responsive) and therefore scored as asymptomatic. Other mouse displays end-point criteria including being unable to stand, displaying rolling behaviour, and has skull distention. As minor altered gait was noted before severity of symptoms reached end-point criteria, this mouse was scored as "neurological symptoms followed by skull distention".
