# Supplementary material for: A CARD9 deficiency mouse model recapitulates human chronic CNS candidiasis identifying defective monocytic cell responses in immunopathogenesis
Source: JCI Insight. 2025 May 27;10(13):e176676. doi: 10.1172/jci.insight.176676 (PMC12288897; doi:10.1172/jci.insight.176676)
Supplement: Unedited blot and gel images [file jciinsight-10-176676-s135.pdf]

Full unedited blot for Figure 1 B

Full unedited gel for Figure 1 B

Western Blot

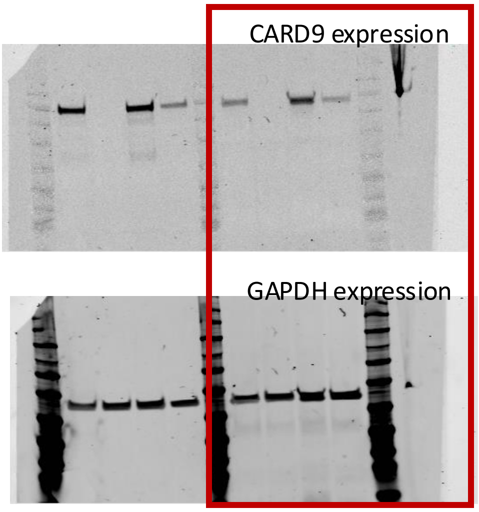

Splenocytes BMDM

RT-PCR Gel

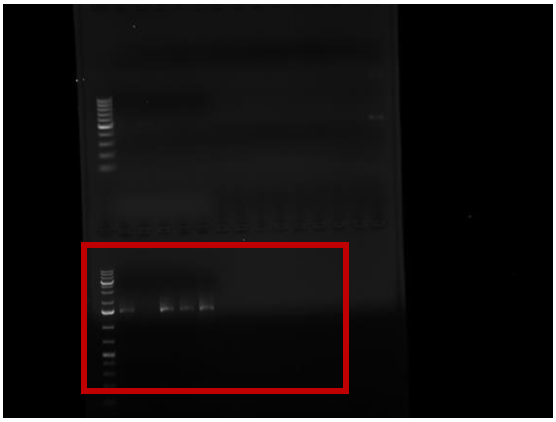

Order loaded: ladder, B6J, CARD9, B6N, Y91HKI, SIM-A9 (microglia cell line expressing CARD9)

CARD9 western blot
Primary: rabbit anti-CARD9 polyclonal antibody (Abnova)
Secondary: Goat anti-Rabbit IgG DyLight 800 (Invitrogen)

GAPDH western blot
Primary: mouse anti-GAPDH antibody (Millipore)
Secondary: Goat anti-Mouse IgG DyLight 680 (Invitrogen)
